# Predicting Agitation Events in the Emergency Department Through Artificial Intelligence

**DOI:** 10.1001/jamanetworkopen.2025.8927

**Published:** 2025-05-07

**Authors:** Ambrose H. Wong, Atharva V. Sapre, Kaicheng Wang, Bidisha Nath, Dhruvil Shah, Anusha Kumar, Isaac V. Faustino, Riddhi Desai, Yue Hu, Leah Robinson, Can Meng, Guangyu Tong, Steven L. Bernstein, Kimberly A. Yonkers, Edward R. Melnick, James D. Dziura, R. Andrew Taylor

**Affiliations:** 1 Department of Emergency Medicine, Yale School of Medicine, New Haven, Connecticut; 2 Department of Biostatistics, Yale School of Public Health, New Haven, Connecticut; 3 Department of Emergency Medicine, Geisel School of Medicine at Dartmouth, Lebanon, New Hampshire; 4 Department of Psychiatry, University of Massachusetts Chan Medical School, Worchester; 5 Section of Biomedical Informatics and Data Science, Yale School of Medicine, New Haven, Connecticut

## Abstract

**Importance:**

Agitation events are increasing in emergency departments (EDs), exacerbating safety risks for patients and clinicians. A wide range of clinical etiologies and behavioral patterns in the emergency setting make agitation prediction difficult in this setting.

**Objective:**

To develop, train, and validate an agitation-specific prediction model based on a large, diverse set of past ED visit data.

**Design, Setting, and Participants:**

This cohort study included electronic health record data collected from 9 ED sites within a large, urban health system in the Northeast US. All ED visits featuring patients aged 18 years or older from January 1, 2015, to December 31, 2022, were included in the analysis and modeling. Data analysis occurred between May 2023 and September 2024.

**Exposures:**

Variables that served as potential exposures of interest, encompassing demographic information, patient history, initial vital signs, visit information, mode of arrival, and health services utilization.

**Main Outcomes and Measures:**

The primary outcome of agitation was defined as the presence of an intramuscular chemical sedation and/or violent physical restraint order during an ED visit. A clinical model was developed to identify risk factors that predict agitation development during an ED visit prior to symptom onset. Model performance was measured using area under the receiver operating characteristic curve (AUROC) and area under the precision recall curve (PR-AUC).

**Results:**

The final cohort comprised 3 048 780 visits. The cohort had a mean (SD) age of 50.2 (20.4) years, with 54.7% visits among female patients. The final artificial intelligence model used 50 predictors for the primary outcome of predicting agitation events. The model achieved an AUROC of 0.94 (95% CI, 0.93-0.94) and a PR-AUC of 0.41 (95% CI, 0.40-0.42) in cross-validation, indicating good discriminative ability. Calibration of the model was evaluated and demonstrated robustness across the range of predicted probabilities. The top predictors in the final model included factors such as number of past ED visits, initial vital signs, medical history, chief concern, and number of previous sedation and restraint events.

**Conclusions and Relevance:**

Using a cross-sectional cohort of ED visits across 9 hospitals, the prediction model included factors for detecting risk of agitation that demonstrated high accuracy and applicability across diverse patient populations. These results suggest that clinical application of the model may enhance patient-centered care through preemptive deescalation and prevention of agitation.

## Key Points

**Question:**

Can artificial intelligence be used to accurately predict agitation events in emergency departments (EDs) prior to their development?

**Findings:**

In this cohort study of 3 048 780 ED visits, an agitation event prediction model including a comprehensive set of predictors was developed that had high accuracy and applicability across diverse patient populations.

**Meaning:**

Knowing key factors associated with agitation may help clinicians in the ED more accurately predict and apply specific deescalation and patient-centered strategies prior to an agitation event.

## Teaser

This cohort study evaluates whether artificial intelligence (AI) can be used to accurately predict agitation events among adult patients visiting the emergency department.

## Introduction

Behavioral and psychiatric conditions in emergency settings are rapidly rising in the US.^1^ Over the past decade, general emergency department (ED) visits for mental health conditions increased by 53%, while overall visits increased by only 8.6%.^2^ Agitation, defined as excessive psychomotor activity leading to aggressive and violent behavior,^3^ is a frequent feature of mental health–related emergency visits. Studies have estimated that as high as 1.7 million episodes of agitation occur annually in EDs across the US,^4^ and 83% of patients presenting with agitation have an underlying serious mental illness.^5^ Once agitation occurs, clinicians must rapidly diagnose potential causes and intervene to minimize harm.^6^ Clinicians in the emergency setting routinely use physical restraints and chemical sedation to care for an agitated patient. However, the use of restraints is associated with up to 37% risk of patient injury, including blunt chest trauma, asphyxiation, respiratory depression, and even sudden death.^7,8,9,10,11,12,13^ To reduce potential patient harm, regulatory bodies, such as The Joint Commission and the Centers for Medicare & Medicaid Services, encourage early identification of patients at risk for agitation and intervention prior to onset of agitation.^14^ This approach aims to prevent progression of agitation symptoms to the point at which deescalation is no longer effective and use of restraints may be needed.

Despite these best practice recommendations, there remains substantial variability in how at-risk patients are assessed and treated in the ED.^15,16,17^ Specific challenges unique to symptoms of agitation that arise in the emergency setting complicate early identification of at-risk patients.^18^ First, agitation is often multifactorial, and underlying causes may include a range of mental health disorders, substance intoxication, and medical comorbidities.^5^ Additionally, knowledge about specific symptoms and risk factors of agitation that would warrant preemptive intervention is limited at best.^18^ As a result, ED clinicians mostly rely on anecdotal experience and expert recommendations to make decisions, potentially causing unnecessary use of restraints if risk of agitation could have been predicted and prevented.^14^ Thus, identifying a set of evidence-based risk factors most predictive of developing agitation could substantially improve the quality and safety of behavioral emergency care.

Prediction models are increasingly being used to complement clinical reasoning and decision-making in modern medicine.^19,20,21,22^ Examples have included applications for cardiology,^19^ diabetes care,^23^ chronic kidney disease,^24^ and hospital readmissions,^22^ but current development of prediction models for mental health has been more limited.^25^ Researchers have developed prediction models for agitation in settings such as the intensive care unit,^26,27^ nursing homes,^28^ and institutionalized dementia units.^10,28^ To our knowledge, a prediction model to identify individuals at risk of agitation in the emergency setting does not exist.

To address this gap, we aimed to develop a novel prediction model using clinical variables routinely available in the ED, including patient characteristics and clinical data, to identify patients at risk of developing agitation. A model that can effectively predict agitation for individuals presenting to the ED may play a crucial role to facilitate timely intervention using evidence-based strategies and minimize the use of unnecessary restraints.^29^

## Methods

### Study Design, Setting, and Participants

This retrospective cohort study followed the Strengthening the Reporting of Observational Studies in Epidemiology (STROBE) guideline^30^ and was conducted using electronic health record (EHR) data to build a prediction model. We included all ED visits between January 1, 2015, and December 31, 2022, for patients aged 18 years or older presenting to 1 of 9 hospital EDs in a regional hospital network in the Northeast US. Hospitals included 2 nonacademic urban, 2 academic urban, and 5 nonacademic suburban sites. To promote generalizability of results and avoid reinforcing misconceptions that agitated behavior may be due only to mental health conditions, we included all visits regardless of associated diagnoses at the ED visit.^31^ The institutional review board of Yale School of Medicine approved this study and waived the need for informed consent due to minimal risk.

### Data Collection and Preprocessing

All data elements for each ED visit were obtained from the enterprise data warehouse. For variables to be included, we first identified a list of potential risk factors (eTable 1 in Supplement 1) for developing agitation in the ED based on (1) existing literature on risk factors for agitation,^3,29,32^ (2) our framework of ED agitation based on staff perspective of agitation management,^33^ and (3) prior studies by members of our team on patient experiences, physical restraint and sedation use, and direct observations of agitation events.^34,35^ Next, we performed an expert-guided variable screening process and excluded several categories of variables to improve the model’s relevance and predictive power. These excluded variables encompassed those deemed irrelevant or with poor data quality. For instance, variables such as time to restraint (98.29% missing) were removed because they were either rarely recorded or incomplete across sites. To maintain computational efficiency and model interpretability, variables with excessive granularity were consolidated where necessary. For instance, the variable drug type initially contained 91 unique drug entries, which were categorized into broader groups, including opioids, stimulants, depressants, hallucinogens, cannabis, other, and unknown (for missing values), to ensure meaningful representation while preserving its relevance to the restraint outcome.

Categorical variables were one-hot encoded to enable their inclusion in the modeling process. Chief concerns were manually recategorized into 19 broader categories from over 1200 unique entries to enhance data clarity and ensure the relevance of the variable for predicting agitation (eAppendix in Supplement 1). Medical history and previous visit diagnoses were matched with Agency for Healthcare Research and Quality Clinical Classifications Software Refined groupings.^36^ For outpatient medications, we used the Anatomical Therapeutic Chemical Classification System to sort and group them into meaningful medication categories.^37^ For continuous variables exhibiting outliers, consisting of blood pressure readings (systolic, diastolic), respiratory rate, and oxygen saturation, extreme values were trimmed at the 1st and 99th percentiles. This approach preserved data points indicative of potential agitation without allowing for the most extreme, potentially spurious values to distort the analysis. Age values beyond 110 years were capped to prevent skewness from unrealistic entries. Columns that might introduce bias or act as proxies were removed to enhance model validity. Time-related variables, including the length of hospital stay, as well as proxy indicators such as types of restraint and disposition outcomes, were omitted from the final model. Although race and ethnicity were initially excluded to avoid introducing bias, these variables were retained for future fairness assessments, ensuring that the model’s performance could be evaluated across demographic groups. As with all variables included in the analyses, race and ethnicity were extracted directly from the EHR as entered during the encounter. Classifications may represent self-identification or assignment by hospital registration. Options for race consisted of American Indian or Native American, Asian, Black, Native Hawaiian or Pacific Islander, White, other, or unknown; options for ethnicity consisted of Hispanic or Latino, non-Hispanic, and unknown. Other race was inclusive of not listed and other races and ethnicities as listed choices within the health record.

The final list of predictor variables included demographic information (eg, age, insurance, employment status, and financial status), patient history (eg, psychiatric and medical), initial vital signs, visit information (eg, chief concern, outpatient medications, and drug type), and health services utilization (eg, presence of a primary care clinician). Data were preprocessed according to the aforementioned methods. Errant text data in categorical fields were improved through regular expression searches. Medications and comorbidities were grouped using the Anatomical Therapeutic Chemical Classification System and Clinical Classifications Software Refined categories. Missing values for categorical variables were coded as unknown or assigned an appropriate category. A total of 682 variables were entered into the selection process (eTable 2 in Supplement 1).

### Target Outcome

Our primary outcome of agitation was defined as the presence of a violent physical restraint order and/or an order for an intramuscular chemical sedative during the ED visit. Violent restraints were indicated for management of behavior that jeopardized the immediate physical safety of the patient, staff, or others as defined by The Joint Commission standards.^38^ Orders for nonviolent restraints for protection of equipment or promotion of medical healing, such as preventing self-extubation, were not included in the primary outcome. Violent restraints were broadly defined by their intent of use for violent behavior in behavioral emergencies and may encompass different types of equipment or restraints that overlap with equipment used in nonviolent restraints. Sedatives were inclusive of medications defined as used for chemical sedation in emergency settings in the literature: antipsychotics (haloperidol, droperidol, olanzapine, aripiprazole, and ziprasidone), benzodiazepines (lorazepam, midazolam), ketamine, and antihistamines (diphenhydramine, hydroxyzine; antihistamines were only included when given in conjunction with an antipsychotic).^39,40^ Prior observations and analysis of the dataset by members of our team^41,42^ have confirmed that sedation and restraint orders were placed with higher than 99% of compliance by the ED clinicians in this study (A.H.W., S.L.B., E.R.M., and R.A.T.).

### Feature Selection, Model Development, and Methods of Assessment

To optimize model performance, we conducted a thorough feature selection process using several complementary techniques. These included recursive feature elimination (RFE), L1-regularized logistic regression, random forest feature importance, and gradient-boosted decision tree (GBDT) (Extreme Gradient Boosting [XGBoost]; Nvidia) feature importance.^43^ Additionally, Shapley additive explanations (SHAP) values were used to gain further insight into the contribution of individual features. RFE was first applied using a logistic regression model with L1 regularization, systematically removing less critical variables until the optimal subset was identified. L1-regularized logistic regression was then used independently to select features based on their coefficients. Random forest and GBDT models provided additional feature rankings through their respective importance scores. SHAP values from the GBDT model offered interpretability, allowing us to assess the influence of each feature on the model’s predictions. A total of 682 variables were initially considered for selection. Following this multistep process, we identified and evaluated the top 20, 50, 100, and 200 features. A consensus was reached by aggregating the most predictive features across all methods, allowing us to refine the final feature set for model development.

The derivation study sample included 8 of the 9 sites and was randomly divided into a training cohort (75%) and a testing cohort (25%) to estimate the probability of an agitation event using logistic regression. Three supervised machine learning models—random forest, least absolute shrinkage and selection operator (LASSO), and GBDT—were used to address potential issues of overfitting, collinearity, and sparse data bias.^44^ A 5-fold cross-validation was applied to each model to determine optimal hyperparameters. For the random forest model, parameters such as the number of trees, maximum depth, and minimum samples per split were tuned. In the case of the GBDT model, learning rate, maximum depth, and regularization terms were optimized. LASSO regression involved tuning the regularization strength (L1) and tolerance. Hyperparameter tuning for all models was conducted using the tree-structured Parzen estimator algorithm, with the best parameters selected based on the minimization of cross-validation error measured by the area under the receiver operating characteristic curve (AUROC) and the area under the precision-recall curve (PR-AUC). We primarily used the AUROC metric to select the best-performing hyperparameters, as it provided a broad measure of overall discriminative ability and ensured a consistent comparison across models. While PR-AUC was also valuable in imbalanced contexts, we opted for AUROC for uniformity. To determine which hyperparameters performed best, we used 5-fold cross-validation for each combination of feature subset (eg, 20, 50, 100, 200, or all 694 features) and model type (random forest or logistic regression). For each combination, we computed the mean AUROC across the 5-folds and chose the set of hyperparameters that yielded the highest mean score.

We recognized that optimizing strictly for AUROC or PR-AUC might risk overfitting, so we used several safeguards. Hyperparameter tuning took place entirely within the cross-validation framework, reducing the likelihood of overfitting to a single fold. In addition, regularization techniques, such as LASSO for logistic regression, and constraints, such as min_samples_split, min_samples_leaf, and max_depth for the random forest, helped control model complexity.

After hyperparameter tuning, the models’ performance was evaluated on both the validation and the external test datasets to assess their generalization capabilities. Feature selection was conducted using RFE to identify the top 20, 50, 100, and 200 predictive features. Each subset of features was evaluated across the random forest, GBDT, and LASSO models, with comparisons based on AUROC and PR-AUC metrics. Ultimately, the GBDT model as a standalone with 50 features was selected as the best-performing model based on these evaluations.

Model performance was further assessed using metrics such as AUC, precision, recall, and F1 score. Calibration of the models was evaluated using calibration curves comparing predicted probabilities with actual outcomes. A perfectly calibrated model would align closely with a 45-degree diagonal line, indicating an ideal correspondence between predicted and observed outcomes. Calibration performance was visualized by plotting the mean predicted probability against the fraction of positive outcomes across multiple bins.

Next, we externally validated the model using a set of 630 000 ED visits from a nonacademic urban hospital within the Yale New Haven Health System (a site not included in the derivation dataset) within the same time interval. This urban site offered a notably broad demographic representation and had distinct clinical characteristics compared with the remaining sites in the derivation cluster. These differences made it an ideal testing ground to evaluate how well the model would perform in a population setting that diverged from the original training cohort. To ensure that the models performed equitably across different subgroups, we conducted a detailed fairness assessment based on age, sex, and racial and ethnic groups. For each subgroup, model performance was evaluated using key metrics, including the AUROC, PR-AUC, and calibration metrics. Given the imbalanced nature of our data, we focused on the AUROC and PR-AUC, as these metrics more effectively captured performance than accuracy alone. Calibration was assessed through bootstrapping to estimate 95% CIs for intercepts and slopes, allowing us to gauge overestimation or underestimation in predicted probabilities. This analysis quantified potential biases in predictions across different groups, with results reported alongside 95% CIs to ensure statistical robustness.

### Statistical Analysis

Baseline characteristics of the study sample were described using frequency and percentage, mean and SD, or median and IQR. Statistical analyses were performed using R, version 4.3.2 (R Project for Statistical Computing). Additional analyses, including model development and testing, were conducted in Python, version 3.12.5. All tests were 2-tailed, and *P* < .05 was considered significant. Data analysis for the study occurred between May 2023 and September 2024.

## Results

### Cohort Characteristics and Data Processing

**Figure 1.**
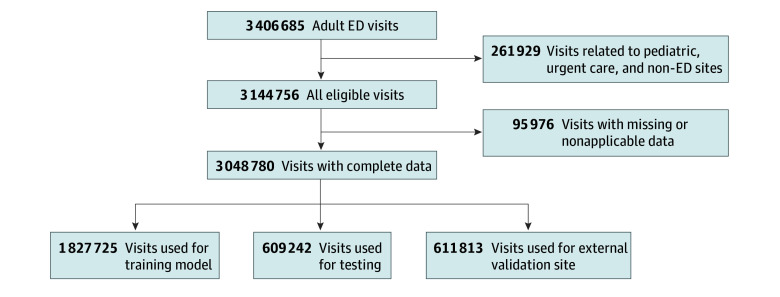
Flow Diagram for Emergency Department (ED) Visits Included in the Study and Breakdown of Visits Used for Training, Testing, and External Validation

From a total of 3 406 685 ED visits screened for inclusion, 261 929 (7.7%) were excluded due to visits at non-ED sites, as shown in Figure 1. An additional 95 976 visits (2.8%) were excluded due to missing or nonapplicable data. The final analytic cohort comprised 3 048 780 visits, with 2 436 967 visits from the derivation dataset across 8 EDs and the remaining encounters forming the independent validation dataset from a single ED. The analytic cohort had a mean (SD) age of 50.2 (20.4) years, with a distribution of 1 666 473 visits among females (54.7%) and 1 379 929 among males (45.3%). A total of 11 623 visits (0.4%) were among American Indian or Native American individuals; 41 700 (1.4%), among Asian individuals; 417 913 (13.7%), among Black individuals; 10 089 (0.3%), among Native Hawaiian or Pacific Islander individuals; 1 737 681 (57.0%), among White individuals; and 729 081 (23.9%), among those reporting other race. Overall, 640 808 visits (21.0%) were among Hispanic or Latino individuals and 2 383 839 (78.2%) among non-Hispanic individuals (Table 1).

**Table 1.** Basic Descriptive Statistics of Electronic Health Record Datasets Used for the Prediction Model of Agitation, January 1, 2015, to December 31, 2022

Characteristic	Emergency department visits, No. (%)					
	Training		Testing		External validation	
	Nonevents (n = 1 809 080)	Events (n = 16 840)	Nonevents (n = 603 414)	Events (n = 5596)	Nonevents (n = 605 705)	Events (n = 5796)
Events, %	NA	0.9	NA	0.9	NA	1.0
Age, mean (SD), y	51.2 (20.7)	44.6 (17.5)	51.2 (20.7)	44.1 (17.1)	48.3 (20.0)	48.8 (20.5)
Sex						
Female	985 223 (54.5)	6805 (40.4)	329 278 (54.6)	2238 (40.0)	340 452 (56.2)	2477 (42.7)
Male	823 842 (45.5)	10 035 (59.6)	274 129 (45.4)	3358 (60.0)	265 246 (43.8)	3319 (57.3)
Unknown	15 (<0.1)	0	7 (<0.1)	0	7 (<0.1)	0
Race						
American Indian or Native American	7714 (0.4)	68 (0.4)	2573 (0.4)	22 (0.4)	1231 (0.2)	15 (0.3)
Asian	27 523 (1.5)	134 (0.8)	9183 (1.5)	43 (0.8)	4792 (0.8)	25 (0.4)
Black	401 562 (22.2)	4859 (28.8)	133 794 (22.2)	1582 (28.3)	185 335 (30.6)	1949 (33.6)
Native Hawaiian or Pacific Islander	6241 (0.3)	50 (0.3)	2113 (0.4)	15 (0.3)	1660 (0.3)	10 (0.2)
White	1 123 392 (62.1)	9628 (57.2)	374 843 (62.1)	3226 (57.6)	222 514 (36.7)	2427 (41.9)
Other^a^	186 125 (10.3)	1557 (9.2)	62 042 (10.3)	523 (9.3)	166 440 (27.5)	1226 (21.2)
Unknown	56 526 (3.1)	544 (3.2)	18 866 (3.1)	185 (3.3)	23 733 (3.9)	144 (2.5)
Ethnicity						
Hispanic or Latino	319 147 (17.6)	2728 (16.2)	106 616 (17.7)	914 (16.3)	209 900 (34.7)	1503 (25.9)
Non-Hispanic	1 475 275 (81.5)	13 945 (82.8)	492 869 (81.7)	4617 (82.5)	392 861 (64.9)	4272 (73.7)
Unknown	14 658 (0.8)	167 (1.0)	4929 (0.8)	65 (1.2)	2944 (0.5)	21 (0.4)

Abbreviation: NA, not applicable.

^a^ Other race was inclusive of not listed and other races and ethnicities as listed choices within the health record.

### Model Development and Performance

**Figure 2.**
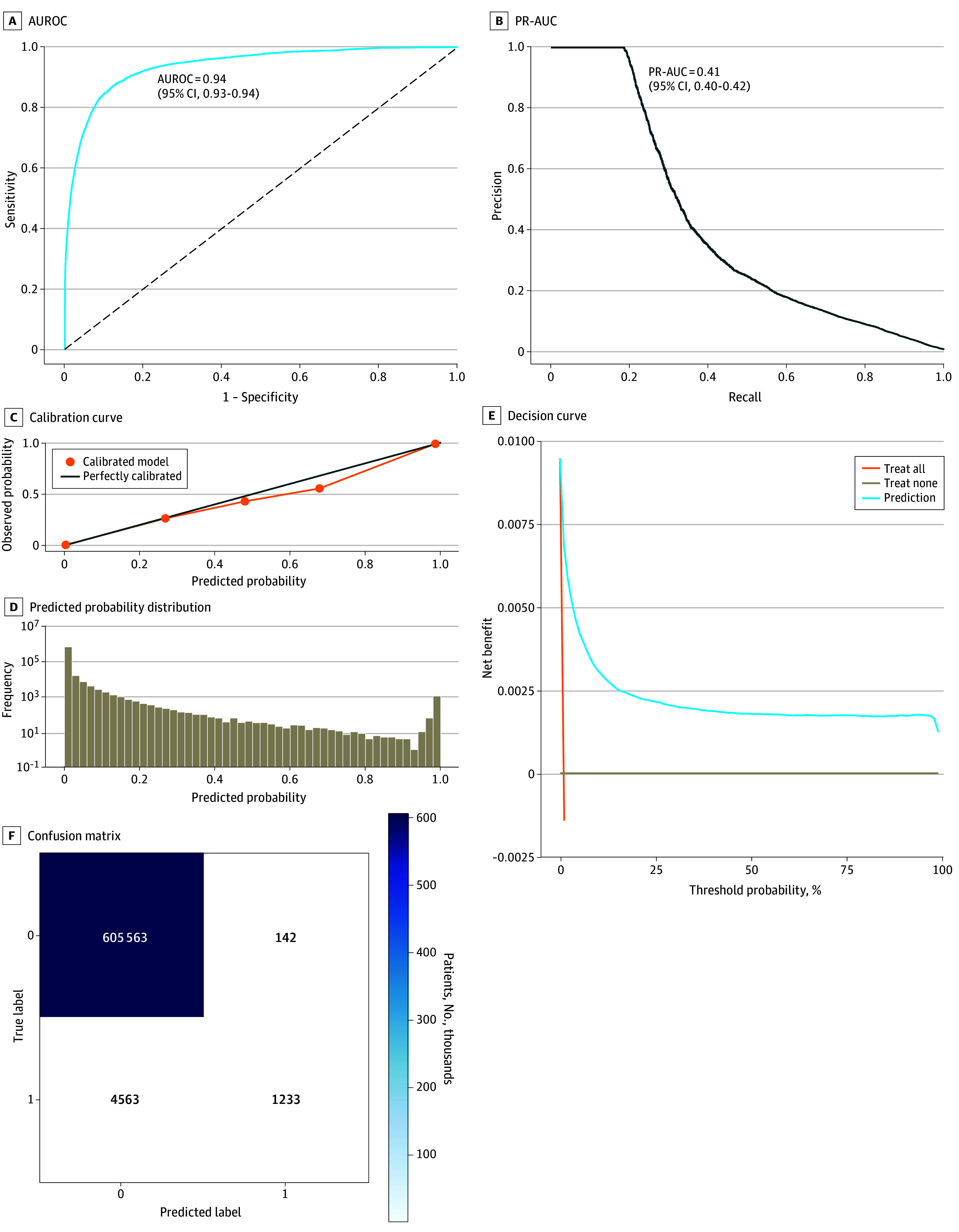
Model Performance and Evaluation Plots for the External Cohort AUROC indicates area under the receiver operating characteristic curve; PR-AUC, area under the precision recall curve.

The artificial intelligence model used 50 predictors for the primary outcome of predicting agitation events, which were defined as the necessity for chemical sedation and/or physical restraint (eTable 3 in Supplement 1 gives the entire list of included features). The model achieved an AUROC of 0.94 (95% CI, 0.93-0.94) and a PR-AUC of 0.41 (95% CI, 0.40-0.42) in the external dataset, indicating good discriminative ability (Figure 2A and B). The validation dataset yielded an AUROC of 0.96 (95% CI, 0.95-0.96) and a PR-AUC of 0.41 (95% CI, 0.41-0.43) (eFigure in Supplement 1). Calibration of the model was evaluated and demonstrated robustness across the range of predicted probabilities (Figure 2C and D). Model evaluation reports for both validation and external datasets are displayed in eTable 4 in Supplement 1.

**Figure 3.**
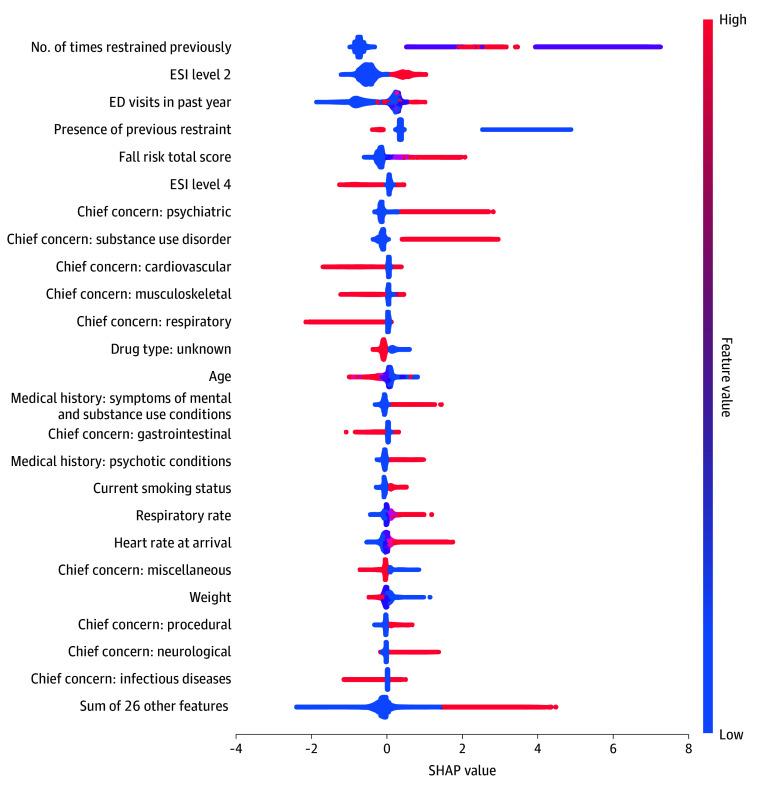
Shapley Additive Explanations (SHAP) Bee Swarm Plot of the Top 25 Predictors of Agitation Events Generated From the Gradient-Boosted Decision Tree Model Each dot corresponds to a single patient observation, and its position along the x-axis indicates how strongly and in which direction the predictor shifted the log-odds of an agitation event. Positive SHAP values (right of 0) indicate that a feature increased the log-odds (and thus the likelihood) of agitation, whereas negative SHAP values (left of 0) show a decrease in the log-odds. ED indicates emergency department; ESI, Emergency Severity Index.

The most important predictors in the final model included factors such as the number of previous ED visits, initial vital signs, medical history, Emergency Severity Index, chief concern, and number of previous sedation and/or restraint events. Detailed coefficients and the relative importance of these predictors are provided in Figure 3.

### Agitation Event Prediction and Validation

In the development dataset, the model identified 0.9% of patients as having risk of experiencing an agitation event. Within visits of patients identified as having agitation, 65.2% had chemical sedation only, 12.8% had physical restraint only, and 22.0% had both physical restraint and chemical sedation. We included a breakdown of agitation visits by individual sedatives used in eTable 5 in Supplement 1. Test characteristics, including sensitivity, specificity, positive predictive value, and negative predictive value, for predicting agitation events across a range of risk thresholds are presented in Table 2. Model performance was also evaluated across different patient subgroups to assess for potential bias. The analysis indicated stable performance across age groups, sex, and race and ethnicity subgroups, with AUROC values consistently above 0.90 and PR-AUCs consistently above 0.35 (eTable 6 in Supplement 1).

**Table 2.** Risk Thresholds for Agitation and Corresponding Sensitivity, Specificity, and Positive and Negative Predictive Values

Agitation risk threshold	Sensitivity	Specificity	Positive predictive value	Negative predictive value
0.10	0.44	0.99	0.30	0.99
0.20	0.31	1.00	0.53	0.99
0.30	0.26	1.00	0.71	0.99
0.40	0.23	1.00	0.83	0.99
0.50	0.21	1.00	0.90	0.99
0.60	0.20	1.00	0.94	0.99
0.70	0.19	1.00	0.98	0.99
0.80	0.19	1.00	0.99	0.99
0.90	0.19	1.00	1.00	0.99
0.98	0.17	1.00	1.00	0.99

### Decision Curve Analysis

The decision curve analysis highlighted the clinical utility of the model across a range of risk thresholds for predicting agitation events. The model demonstrated a higher net benefit compared with both universal screening and no screening approaches across these thresholds, indicating its potential value in clinical settings for early identification and intervention in high-risk patients (Figure 2E and F).

## Discussion

Using EHR data from 3 048 780 ED encounters, this was the first study, to our knowledge, to derive an evidence-based prediction model for risk of developing agitation in the emergency setting. The introduction of artificial intelligence models to predict agitation events in EDs represents potential in the advancement in the fields of psychiatry and emergency medicine. The model’s ability to identify patients at risk of developing agitation may provide evidence of data-driven approaches to enhance patient care and safety. Findings from this study may assist in changing the landscape of agitation research and practice by creating evidence-based criteria for identifying patients at risk for agitation in EDs.

The most important predictors in our model included the number of previous ED visits, initial vital signs, medical history, Emergency Severity Index, chief concern, and number of previous sedation and/or restraint episodes. Our findings are consistent with existing literature that identified historical violence as a predictor of future agitation events.^45^ Although agitation is a broader constellation of symptoms that includes violent or aggressive behavior, previous episodes of restraint and sedation may indicate that the individual is at risk of developing higher levels of agitation. The inclusion of initial vital signs as a predictor emphasizes the importance of physiological states in agitation risk, potentially reflecting underlying medical or psychiatric conditions contributing to the agitated state.^3^

Our model also identified several socioeconomic factors that may be associated with agitation in the ED. Types of community and neighborhoods have been associated with violent or aggressive behavior. Individuals with exposure to violence as a young child or adolescent have been shown to have increased likelihood of reduced educational attainment and lifelong unemployment and increased rates of homelessness and gang involvement.^46^ Prior studies have shown that a history of exposure to violence is associated with worse socioeconomic status, which in turn is associated with increased rates of violent or aggressive behavior.^47^ These findings indicate associations between structural vulnerability and increased risk of agitation, and further research may be needed to identify policy and social interventions outside the ED that can target these structural factors.^48^

Our findings build on and extend previous research in several key ways. First, the comprehensive nature of our model, which includes a wide array of predictors from clinical and environmental factors, offers a more holistic and evidence-based approach to agitation risk assessment compared with expert recommendations and anecdotal evidence that may be more focused on workplace violence prevention or evaluation of clinical etiologies without consideration for detection and prediction of symptom development. This broad approach is critical in the ED setting, where the causes of agitation are multifaceted and can stem from a complex interplay of medical, psychiatric, and environmental factors. Currently, to our knowledge, there are no evidence-based instruments to predict agitation that are specific to the ED environment and population.^49^ Implementation of a prediction model that is tailored for the individual patient may help centralize relevant data to the clinician and display just-in-time information that can be helpful in proactive prevention of agitation symptoms.

Second, previous literature has shown that attribution of agitation as well as management practices to treat symptoms of agitation may be influenced by structural or interpersonal biases against historically marginalized populations.^50^ The model’s robust performance across various patient subgroups (including different age groups, sex, and race and ethnicity categories) with AUROCs consistently above 0.90 and PR-AUCs consistently above 0.35 suggests that it is broadly applicable and does not exhibit substantial bias toward any specific demographic group. This is particularly important in ensuring equitable care and avoiding potential disparities in the management of at-risk patients by inadvertently reinforcing biases in the detection and prevention of agitation symptoms. We recognize that certain features may have uneven distributions across demographic groups and could potentially introduce bias into the model. However, these predictors were included to capture situational factors that impact the risk of agitation events in clinical practice. Our primary goal was to develop a pragmatic model that reflects the complexities of the ED setting, including any contextual elements that may indicate an increased need for early deescalation measures. Further exploration of how these variables interact with patient race and ethnicity and other sociodemographic characteristics may be warranted to fully understand the potential for bias. Future work may include qualitative inquiries with stakeholders to identify where biases may be introduced when clinicians seek to identify those at risk of agitation as well as fairness-focused computational methods to mitigate innate biases to EHR data,^51^ including application of debiasing algorithms.^52^

The clinical implications of our findings are interesting in several ways. By improving identification of patients at high risk for agitation, ED clinicians can better tailor interventions to prevent agitation episodes, potentially reducing the need for sedation or physical restraints and the associated risks to patients and staff. Importantly, the model supports a patient-centered approach to care, focusing on early identification and intervention rather than reactive measures, such as restraint or sedation. This approach aligns with best practices recommended by Project BETA (Best Practices in the Evaluation and Treatment of Agitation) and other guidelines that advocate for the minimization of restraint use through preemptive management of agitation^4^ with deescalation, environmental modification, and other behavioral techniques. Early identification and risk stratification for agitation may be particularly important during times of high volume or acuity when staff have limited time and resources.

Even if used at a relatively lower agitation risk threshold (eg, 0.60-0.70), the prediction model’s high positive and negative predictive values at those thresholds may allow staff to focus attention more accurately on patients at risk for agitation in building a strong rapport and trusting therapeutic relationship, making attempts at deescalation more likely to be effective.^34^ The high discriminative performance (AUROC of 0.94) of our model stemmed from its ability to screen out true-negative instances of agitation, while identification of true positives was lower. Future studies may need to investigate methods to capture additional subgroups with risk for agitation, for example, through additional EHR elements or live clinical data. Additional work will also be needed to allow for clinical implementation of this type of prediction model for agitation. This implementation includes (1) selection of an optimal risk threshold in the clinical ED context to balance precision and recall for patient care (Table 2), (2) engagement with frontline staff and clinicians regarding how best to incorporate detection and prediction into their daily practice and workflow, and (3) coordination with EHR vendors to ensure seamless integration of the prediction model into the electronic workspace.

Our study also addressed some of the limitations observed in previous predictive models for agitation. Unlike models developed in specialized settings, such as intensive care units, or for specific populations, such as patients with dementia, our model was derived from a general ED population and is designed to predict agitation within a narrow time window, enhancing its relevance and applicability to the ED setting. Furthermore, by developing the model on a large, multicenter dataset, we improved its generalizability and potential for implementation across various health care settings.

### Limitations

This study has several limitations. Factors included in the model were derived from data elements extracted from the EHR and may not capture environmental and systems characteristics of an ED visit that may contribute to development of agitation. Other EHR variables that may be incomplete, not collected, or not present in our health system could lead to potential gaps or less power in our prediction model. Potential redundancy in some of our included features may have limited utility; we incorporated RFE procedures to retain features that improved cross-validation performance. Our dataset was from a single health system and may represent local clinical, procedural, restraint or sedation practice–related, or logistical factors, thus limiting generalizability. However, our sites represented a range of settings across a wide geographical area, allowing for variation in types of patient demographics and clinical practice in our study.

## Conclusions

In this cohort study of EHR data, our results presented a substantial advancement in the prediction of agitation events in EDs using artificial intelligence. By leveraging a large dataset and a comprehensive set of predictors, we developed a model that was both accurate and applicable across diverse patient populations. The clinical implementation of this model has the potential to transform the management of agitation in the emergency setting, enhancing patient safety and care while reducing the reliance on chemical sedation and physical restraints. Future research should focus on the applications of this model, including the integration into EHR systems and the assessment of its influence on clinical outcomes and sedation and restraint use in the clinical environment.

## Notes

Supplement 1. **eTable 1.** Potential Risk Factors From the Electronic Health Record That May Predict Development of Agitation in the Emergency Department **eAppendix.** Chief Complaint Categorizations **eTable 2.** A List of All Included Features and Associated Descriptions **eTable 3.** Final List of Features Included in the Model and Associated Shapley Additive Explanations (SHAP) Values **eTable 4.** Model Evaluation Reports for Validation and External Datasets **eTable 5.** List of Sedative Medications and Percentages of Agitation Events That Included Administration of Each Medication **eTable 6.** Fairness Assessment and Model Performance Across Age, Sex, and Race and Ethnicity Categories **eFigure.** Model Performance During Cross-Validation

Supplement 2.
Data Sharing Statement
