# Supplementary material for: Predicting Agitation Events in the Emergency Department Through Artificial Intelligence
Source: JAMA Netw Open. 2025 May 7;8(5):e258927. doi: 10.1001/jamanetworkopen.2025.8927 (PMC12059975; doi:10.1001/jamanetworkopen.2025.8927)
Supplement: Supplement 1. — eTable 1. Potential Risk Factors From the Electronic Health Record That May Predict Development of Agitation in the Emergency Department eAppendix. Chief Complaint Categorizations eTable 2. A List of All Included Features and Associated Descriptions eTable 3. Final List of Features Included in the Model and Associated Shapley Additive Explanations (SHAP) Values eTable 4. Model Evaluation Reports for Validation and External Datasets eTable 5. List of Sedative Medications and Percentages of Agitation Events That Included Administration of Each Medication eTable 6. Fairness Assessment and Model Performance Across Age, Sex, and Race and Ethnicity Categories eFigure. Model Performance During Cross-Validation [file jamanetwopen-e258927-s001.pdf]

Supplementary Online Content

Wong AH, Sapre AV, Wang K et al. Predicting agitation events in the emergency department through artificial intelligence. *JAMA Netw Open*. 2025;8(5):e258927.
doi:10.1001/jamanetworkopen.2025.8927

eTable 1. Potential Risk Factors From the Electronic Health Record That May Predict Development of Agitation in the Emergency Department
eAppendix. Chief Complaint Categorizations
eTable 2. A List of All Included Features and Associated Descriptions
eTable 3. Final List of Features Included in the Model and Associated Shapley Additive Explanations (SHAP) Values
eTable 4. Model Evaluation Reports for Validation and External Datasets
eTable 5. List of Sedative Medications and Percentages of Agitation Events That Included Administration of Each Medication
eTable 6. Fairness Assessment and Model Performance Across Age, Sex, and Race and Ethnicity Categories
eFigure. Model Performance During Cross-Validation

This supplementary material has been provided by the authors to give readers additional information about their work.

© 2025 Wong AH et al. *JAMA Network Open*.

eTable 1. Potential Risk Factors From the Electronic Health Record That May Predict Development of Agitation in the Emergency Department

Domain	Data Elements
**Patient Factors**	**Demographics:** age, preferred language, insurance status, employment status, height, weight, body-mass index (BMI)<br><br>**History:** chief complaint, psychiatric/medical history, alcohol/substance/tobacco use, history of housing insecurity, number of emergency department visits per year, outpatient prescribed medications, presence of behavioral flags, number of previous restraint and/or sedation
**Clinical Data**	**Initial Vital Signs:** heart rate, temperature, systolic/diastolic blood pressures, oxygen saturation, respiratory rate<br><br>**Visit information:** chief complaint, emergency severity index (1-5), arrival month/day/hour, length of stay
**System**	**Pre-Arrival:** mode of transport into ED, presence of law enforcement escort<br><br>**Health Services:** presence of primary care clinician, number of previous hospitalizations

© 2025 Wong AH et al. *JAMA Network Open*.

eAppendix. Chief Complaint Categorizations

Oncologic

Lymphoma, Leukemia, Testicular Cancer, Ovarian Cancer, Prostate Cancer, Skin Cancer, Breast Cancer, Colon Cancer, Pancreatic Cancer, Gastric Cancer, Kidney Cancer, Breast Cancer, Melanoma, Squamous Cell Carcinoma, Lung Cancer, Liver Metastasis, Metastasis, Kidney Cancer, Cancer Pain, Colon Cancer, Brain Tumor, Leukemia, Bladder Cancer, Breast Cancer, Head And Neck Cancer, Mass, Cancer, Breast Mass, Lung Cancer Screening, Chemotherapy, Brain Tumor, Multiple Myeloma, Radiation, Adenopathy, Cervical Cancer, Vulvar Cancer, Vaginal Cancer, CLL (Chronic Lymphocytic Leukemia), Multiple Myeloma, Hepatic Cancer

Drug And Alcohol Related

Withdrawal- Drug, Withdrawal, Opioid Use Disorder, Cocaine Use, Nicotine Dependence, Overdose- Intentional, Addiction Problem, Delirium Tremens (Dts), Heroine Overdose-Intentional, Heroine Overdose-Accidental, Opioid Use Disorder, Alcohol Intoxication, Alcoholic Seizure, Opioid Use Disorder, Overdose- Accidental, Drug Overdose, Opiate Withdrawal, Drug Problem, Alcohol Problem, Withdrawal- Alcohol, Alcohol Use, Alcohol Use Disorder

Nasal/ENT Conditions

Nasal Deformity, Ear Problem, Nasal Swelling, Ear Swelling, Nasal Swelling, Cerumen Impaction, Hearing Loss, Ringing In Ear, Nasal Polyps, Earache, Nasal Drainage, Nasal Injury, Ear Re-Evaluation, Nasal Congestion, Hearing Problem

Ophthalmic Conditions

Eye Irritation, Eye Drainage, Tearing, Eye, Visual Disturbance, Eyelid Mass, Iritis, Spots And/Or Floaters, Itchy Eye, Decreased Visual Acuity, Eye Burn, Glaucoma, Vision Change, Visual Field Change, Uveitis, Eye Trauma, Keratitis, Eye Re-Evaluation, Diplopia, Macular Degeneration, Eye Swelling, Eye Puncture Wound, Burning Eyes, Red Eye, Dry Eye, Eye Injury, Photophobia, Eye Itching, Flashes, Light, Flashes, Blepharitis, Cornea Check, Foreign Body In Eye, Eye Lesion, Eye Strain, Loss Of Vision, Anisocoria, Eye Pain, Eye Discharge, Irregular Eye Movement, Eye Burn, Diplopia, Foreign Body In Eye, Eye Pain, Eye Discharge, Irregular Eye Movement, Eye Burn, Itchy Eye, Spots, Burning Eyes, Myopia, Stye, Epiretinal Membrane, Detached Retina, Eye Redness, Pressure Behind The Eyes, Cataract, Blurred Vision, Eye Problem, Amblyopia, Blindness, Visual Disturbance, Decreased Visual Acuity, Loss Of Vision, Lid Droop, Eye Twitching, Cognitive Changes, Cloudy Vision, Sinusitis, Ear Fullness, Tinnitus, Lid Droop, Eye Twitching, Cloudy Vision, Contact Lens Irritation, Ptosis, Anopthalmos, Conjunctivitis, Ciliary Dyskinesia

Urological And Reproductive Conditions

Hydronephrosis, Vaginal Bleed-Pregnant, Ovarian Cyst, Penis Swelling, Genital Itching, Prostate Nodule, Vaginal Swelling, Testicular Cancer, Scrotal Pain, Imminent Delivery, Decreased Fetal Heart Tones, Vaginitis, Menometrorrhagia, Std Exposure, Urinary Catheter Check, Dysuria, Incontinence, Polyuria, Nephropathy, Male Gu Problem, Ectopic Pregnancy, Contractions, Vaginal Discharge 3rd Trimester Pregnancy, Uterine Prolapse, Dyspareunia, Gynecology Problem, High Risk Gestation, Prostate Nodule, Vaginal Bleeding, Vaginal Prolapse, Priapism, Breast Discharge, Pelvic Inflammatory Disease, Perineal Swelling, Nephrolithiasis, Urinary Symptoms, Swollen Breast, Postpartum Complications, Vaginal Bulging Or Protrusion, Difficulty Urinating, Urinary Catheter Insertion Or Check, Urinary Catheter Check, Vaginal Itching, Pre-Eclampsia, Benign Prostatic Hypertrophy, Female Gu Problem, Foreign Body In Penis, Conception, Flank Swelling, Breast Cysts, Mastitis, Pregnancy Ultrasound, Nipple Discharge, Urinary Frequency, Testicle Injury, Nephropathy, Prostate Check, Ovarian Cancer, Routine Prenatal Visit, Menorrhagia, Voiding Issue, Menorrhagia, Urinary Symptoms, Urinary Leakage With Cough/Sneeze/Exercise, Testicle Swelling, Pelvic Pain, Endometriosis, Threatened Miscarriage, Rupture Of Membranes, Perineal Swelling, Prostate Cancer, Menopause,

Dysmenorrhea, Ovarian Cyst, Pelvic Pain, Endometriosis, Polyuria, Hyperemesis Gravidarum, Fibroids, Vaginal Discharge, High Risk Gestation, Pelvic Pain-Pregnant, Recurrent Miscarriage, Anuria, Erectile Dysfunction, Tampon Removal, Scheduled C-Section, Galactorrhea, Bartholin's Cyst, Testicle Pain, Menstrual Problem, Morning Sickness, Menometrorrhagia, Proteinuria, Urinary Urgency, Bladder Infections, Galactorrhea, Vaginal Bleed-Pregnant, Fibroids, Suprapubic Pain, Abnormal Penile Curvature, Bladder Pain, Irregular Menses, Penile Discharge, Pregnancy Problem, Vaginal Pain, Amenorrhea, Pregnancy Us, Dysmenorrhea, Postpartum Care, Std Check, Urinary Retention, Urinary Tract Infection, Urinary Catheter Problem, Vaginal Bleeding- 7 Years Or Less, Possible Pregnancy, Vaginal Bleeding- 7 Years Or Less, Bladder Cancer, Penis Pain, Urethral Discharge, Urinary Incontinence (Stress), Breast Mass, Unprotected Sex, Inability To Postpone Urination, Perineal Laceration, Genital Warts, Umbilical Hernia, Pelvic Mass, Hematuria, Groin Swelling, Foreign Body In Vagina, Ingional Hernia, Fetal Abnormality, Miscarriage, Laboring, Fetal Demise, Late Period, Missed Abortion, Sexual Problem, Sti Check, Sperm Prep, Cystocele, Testicular Mass, Abnormal Pap Smear, Evaluation Follow-Up Living Donor Kidney, Vaginal Cancer, Gynecologic Exam, Premenstrual Syndrome, Prenatal Assessment, Perineal Injury, Vaginal Atrophy, Cervical Cancer, Vulvar Cancer, Loss Of Libido, Enuresis, Metrorrhagia, LEEP

Mental Health
Homicidal, Paranoid, Full Trauma, Schizophrenia, OCD, Dementia, Bipolar, Relationship Issues, Psychosis, Psychotic Symptoms, Anorexia, Behavioral Concern, Self Mutilation, Stress, Panic Attack, Suicide Attempt, Depression, Behavior Problem, Psychiatric Evaluation, Confusion, Irritability- 24 Months Or Less, Alzheimer Dementia, Alleged Domestic Violence, Delusional, Irritability- 24 Months Or Less, Personal Problem, Runaway, Psychotic Symptoms, Alzheimer Dementia, Alleged Domestic Violence, Delusional, Unresponsive, Personal Problem, Runaway, Hallucinations, Mental Health Problem, Anxiety, Suicidal, Stress, Schizophrenia, Manic Behavior, Cognitive Changes, Psychotherapy, Counseling, Alleged Child Abuse

Agitation
Combative, Aggressive Behavior, Agitation

Injuries
Spider Bite, Head Injury With Loc, Burn Re-Evaluation, Head Laceration, Toe Injury, Arm Injury-Major, Leg Injury, Gun Shot Wound, Foot Burn, Toe Injury- Major, Body Laceration, Chest Injury-Major, Swelling At Surgical Site, Ear Injury, Fracture, Head Puncture Wound, Nursemaid's Elbow Injury, Motor Vehicle Crash, Follow-Up Laceration, Vaginal Injury, Crush Injury, Abrasion, Neck Puncture Wound, Shoulder Injury- Major, Chest Injury, Clavicle Injury, Wrist Problem, Facial Puncture Wound, Motorcycle Crash- Major, Diving Accident, Fishhook Removal, Abrasion, Fall-Major, Hip Problem, Spine Injury, Wrist Swelling, Foot Injury, Flank Injury, Orbital Fracture, Clavicle Injury-Major, Foot Laceration, Automobile Vs Pedestrian-Major, Hand Injury- Major, Foot Injury-Major, Abrasion-Complicated, Hip Injury-Major, Facial Burn, Lightning Strike, Ingrown Toenail, Airplane Crash, Motorcycle Crash, Concussion, Puncture Wound-Complex, Finger Injury-Major, Clavicle Swelling, Facial Injury, Head Injury With Unknown Loc, Trauma- Major, Nail Bed Injury, Injury-Major, Testicle Injury, Ankle Injury, Arm Laceration, Human Bite, Shoulder Injury, Back Injury, Arm Injury, Farm Accident, Atv Crash, Intraventricular Hemorrhage, Boat Crash-Major, Finger Injury, Ankle Swelling, Modified Trauma, Ear Laceration, Coccyx Injury, Cat Bite, Snake Bite, Leg Injury- Major, Neck Puncture Wound -Complex, Knee Injury, Finger Swelling, Splint Removal, Nasal Swelling, Fingernail Avulsion, Jaw Injury, Clavicle Pain, Smoke Inhalation, Rib Injury, Thumb Injury- Major, Head Injury, Burn, Facial Burn, Shoulder Problem, Bee Sting, Foreign Body In Nose, Foreign Body In Throat, Upper Extremity Pain, Work-Related Injury, Trauma (Full Trauma), Torticollis, Subungual Hematoma, Stubbed Toe, Smoke Inhalation, Automobile Versus Pedestrian, Wrist Injury, Human Bite, Leg Injury- Major, Neck Puncture Wound -Complex, Finger Swelling, Splint Removal, Nasal Swelling, Fingernail Avulsion, Jaw Injury, Clavicle Pain, Smoke Inhalation, Rib Injury, Thumb Injury- Major, Airway Obstruction, Wound Hematoma, Major Joint Inj/Hip, Shoulder Problem, Foreign Body In Nose, Foreign Body In Throat, Upper Extremity

© 2025 Wong AH et al. *JAMA Network Open*.

Pain, Work-Related Injury, Torticollis, Bicycle Crash-Major, Ankle Injury-Major, Hip Injury, Motor Vehicle Crash-Pregnant, Buttox Injury, Genital Injury, Thumb Injury, Cervical Spine Injury, Bicycle Crash, Electrical Burn, Electric Shock, Rib Swelling, Extremity Laceration, Facial Puncture Wound Complex, Leg Problem, Penis Injury, Chemical Burn, Rodent Bite, Ankle Problem, Elbow Injury, Neck Injury, Modified Trauma, Firework Injury, Bite, Cast Problem-Major, Cast Problem, Laceration, Hand Laceration, Head Injury With Loc, Facial Laceration, Cast Repair, Elbow Injury-Major, Facial Laceration, Jet Ski Crash, Atv Crash-Major, Swallowed Foreign Body, Whiplash, Stab Wound, Hand Burn, Foreign Body In Ear, Hematoma, Wound Hematoma, Wound Check, Head Swelling, Puncture Wound, Injury Or Bleeding Event, Abdominal Injury, Body Laceration, Fall, Fall Greater Than 65 Years Old, Black Eye, Knee Injury-Major, Arm Injury, Taser Injury, Hand Injury, Wrist Injury- Major, Lip Laceration, Knee Injury, Sternum Injury, Airplane Crash, Snake Bite- Venomous, Electrical Burn, Electric Shock, Motorcycle Vs Pedestrian-Major, Motor Vehicle Crash-Major, Burn-Major, Multiple Falls, Dislocation, Injury, Perineal Burn, Motorcycle Vs Pedestrian, Boat Crash, Splinter, Thermal Burn, Tailbone Pain, Skiing Accident, Skiing Accident, Finger Pain, Thumb Swelling, Ankle Pain, Dental Injury, Multiple Lacerations, Wound Dehiscence, Groin Injury, Perineal Contusion, Abdominal Laceration, Sled Injury

Infectious Diseases

Followup Rabies Vaccine, Infectious Exposure- Needle Stick, Recurrent Sinusitis, Frequent Infections, Lyme Disease, Rabies Exposure, Isolation Infectious Disease, Cystitis, Exposure To Std, Tick Bite/Removal, Infectious Exposure-Needle Stick, Fever Immunocompromised, Sinus Problem, Thrush, Lyme Disease, Infections, Isolation Infectious Disease, Exposure To Std, Varicella, Coxsackie Virus, Exposure Coronavirus(Covid-19), Exposure Coronavirus(Covid-19), Pyelonephritis, Tick Removal, Confirmed Coronavirus(Covid-19), Covid-19 Infection, Positive Ppd, Infestations, Sepsis Alert, Covid-19 Screening, Covid-19 Return To Work, Hiv Positive/Aids, Chicken Pox Exposure, Blood Infection, Head Lice, Infection, Insect Bite, Herpes Simplex Virus, Prosthetic Joint Infection, Otitis Media, Blood Infection, Recurrent Skin Infections, Lice, Impetigo, Herpes Zoster, Coxsackie Virus, Tetanus, Lice, Impetigo, Pin Worms, Diarrhea-Pediatric, Herpes Zoster, Coxsackie Virus, Seizures, Cellulitis, Wound Infection- Complicated, Ear Drainage, Influenza, Tuberculosis Exposure, Pin Worms, Otitis Externa, Follow-Up Cellulitis, Possible Exposure To Coronavirus, Dental Abscess, Fever, Suspected Coronavirus(Covid-19), Neutropenia, Infectious Disease, Scabies, Possible Exposure To Coronavirus, Lice, Tuberculosis Exposure, Abscess-Complicated, Hepatitis Exposure, Abscess, Blood Infection, Jaundice, Wound Infection, Fifth Disease, Exposure Coronavirus(Covid-19, Body Fluid Exposure, Fever-8 Weeks Or Less, Recurrent Otitis, Exposure Coronavirus(Covid-19), Subacute Bacterial Peritonitis, Tuberculosis, Anthrax Exposure, Prep (Pre-Exposure Prophylaxis), Evaluation, Known Covid 19

Dermatological Conditions

Hair/Scalp Problem, Blister, Skin Lesion, Scar, Skin Ulcer, Bunions, Mouth Pain, Lip Dryness, Skin Discoloration, Skin Tags, Sunburn, Skin Irritation, Corns, Scar, Blister, Skin Lesion, Skin Discoloration, Rash, Cellulitis, Hair/Scalp Problem, Bunions, Rosacea, Acne, Skin Cancer, Skin Problem, Seborrheic Dermatitis, Facial Burn, Tinea, Ingrown Toenail, Sunburn, Insect Bite, Skin Lesion, Skin Lesion With Prolonged Bleeding, Keloid, Poison Sumac, Recurrent Skin Infections, Melanoma, Varicose Veins, Eczema, Cellulitis, Eczema, Lip Swelling, Plantar Warts, Spots, Spots, Skin Discoloration, Cerebral Palsy, Eczema, Cellulitis, Impetigo, Plantar Warts, Spots, Psoriasis, Dermatitis, Nail Problem, Squamous Cell Carcinoma, Verrucous Vulgaris, Tinea Pedis, Foot Ulcer, Alopecia, Urticaria, Skin Lesion With Prolonged Bleeding, Rosacea, Acne, Plantar Fasciitis, Calluses, Poison Ivy, Split, Diaper Rash, Split, Nevus, Burn-Major, Total Body Skin Exam, Thermal Burn, Sunburn, Poison Oak, Foreign Body In Skin, Epidermal Cyst

Procedures And Follow-Ups

Incision And Drainage Re-Evaluation, Staple Removal, Scheduled Induction, Consult, Post-Op Follow-Up, Discharge Instructions Visit, Referral For Psychiatric Evaluation Txp, Evaluation, Dialysis Shunt Problem, Medical Problem Re-Evaluation, Ob Problem Re-Evaluation, Drainage From Incision, Staple Removal, Incision And Drainage Re-Evaluation, Liver Transplant, Procedure, Follow-Up, Appointment, Total Body Irradiation, Auto Stem Cell Collection, Scheduled

Induction, Imminent Delivery, Post-Op Follow-Up, Discharge Instructions Visit, Cast Re-Evaluation, Ostomy Problem, Follow-Up Medical Evaluation Hepatobiliary, Ventriculoperitoneal Vp Shunt Malfunction, Eeg, Trigger Point Inj-Intercostal Nerve Block,Multiple, Cpap, Evaluation Of Abnormal Diagnostic Test, Evaluation Of Abnormal Diagnostic Test, Blood Work, Epidural, Blood Or Clot Patch, Tracheostomy Tube Change, Postpartum Complications, Assessment For Placement, Therapeutic Plasma Exchange, Dialysis, Dressing Change, Mri, Follow-Up Psychiatric Txp, Suture / Staple Removal, Sleep Apnea Evaluation, Medication Reaction, Ppd Reading, Medication Administration Only, Medication Problem, Iv Medication, Follow-Up Visit, Portacath Problem, Follow-Up Burn, Blood Draw, Medication Management, Follow-Up Medical Problem-Minor, Follow-Up Abscess, Follow-Up Post-Implantation Vad, Follow-Up Psychiatric Txp, Follow-Up Medical Evaluation Hepatobiliary, Follow-Up Burn, Kidney Transplant Follow-Up, Dialysis-Asymptomatic, Splint Removal, Post-Op Problem- Major, Bam Infusion, Medication Management, Follow-Up Medical Problem-Minor, Follow-Up Abscess, Follow-Up Post-Implantation Vad, Follow-Up Psychiatric Txp, Follow-Up Medical Evaluation Hepatobiliary, Follow-Up Burn, Kidney Transplant Follow-Up, Dialysis-Asymptomatic, Splint Removal, Post-Op Problem- Major, Surgical Evaluation, Therapeutic Phlebotomy, Drain Check, Infusion, Scheduled C-Section, Diabetic Follow Up, Tb Risk Assessment, Electric Shock, Annual Exam, Surgical Evaluation, Wound Re-Evaluation, Ultrasound, Emergency Contraception, Gi Tube Problem, Feeding Tube Change, Post-Op Problem, Medical Problem, Radiation Therapy Consult, Elderly Protective Services Eval, Follow-Up Hypertension, Cast Problem, Sedation, Follow-Up Cellulitis, Follow-Up Hypertension, Medical Screening, Pre-Op Exam, Emergency Room Follow Up, Emesis During Pregnancy, Shunt Problem, Emergency Room Follow-Up, Triage Medical Complaint, Ct Scan, Kidney Transplant, Non-Stress Test, Post-Op Infection, Post-Op Problem, Sexual Assault Exam Referral, Or Check, Suture / Staple Removal, Follow-Up Burn, Wound Check, Follow-Up Post-Implantation Vad, Toxicology Screen, Pregnancy Test, Anticoagulation, Medication Inquiry, Radiation Therapy Follow Up, Ostomy Problem, Follow Up After Pacemaker Implant, Defibrillator Check, Surgical Problem Re-Evaluation, Cast Removal, Blood Work, Results, Consultation, Lab Testing, Routine Exam, Transport, Transport Transfusion, Consult, Staple Removal, Medication Inquiry, Problem Visit, Bone Marrow Transplant, On Treatment Visit, Mri, Follow-Up Visit, Discharged, Admission, Iv Hydration, Radiation Therapy Consult, Abdominal Pain Re-Evaluation, Njections, Epistaxis Re-Evaluation, Evaluation For Positive Culture, Flu Vaccine, Wound Drain Evaluation, Post Diagnosis Test Re-Evaluation, Screening, Medication Question, Establish Care, Medication Refill, Dose Adjustment, Covid Related Newborn Check, Feeding Tube Problem, Dental Filling, Referral, Detox Evaluation, Employment Physical, Lymphadenopathy, Sonohystogram, Labs Only, Groshong Or Hickman Line Problem, Injections, Home Health Certification, Referral For Medical Evaluation Heart Transplant, Transfusion, Medical Clearance, Relapse Prevention Follow-Up, Forms, Allo Stem Cell Collection, Therapeutic Apheresis, Groshong Or Hickman Line Problem, Picc Line Problem, Indwelling Line Problem, Medication Adherence, Sweat Test, Pancreas Transplant, Hpc (A) Autologous, Hemoglobin A1c, Post-Op, Diabetic Foot Evaluation, Leep (Loop Electrosurgical Excision Procedure), Echo (Echocardiogram), Anticoagulation Management, Medication Dose Change, Temperature Check, Nephrectomy, Dilation, Genetic Evaluation, Pain Follow-Up, Abr, Dry Socket Post Op

Gastrointestinal Conditions

Abdominal Cramping, Dysphagia, Food Intolerance, Pancreatitis, Gi Tube Evaluation, G-Tube Displacement, Gi Problem, Abdominal Pain, Nausea, Rectal Urgency, Bloating, Abnormal Imaging, Hiatal Hernia, Colon Cancer, Flank Pain, Gastric Mass, Rectal Bleeding-Major, Rectal Bleeding-Minor, Sore Throat- Complicated, Bloody Diarrhea, Ascites, Gastroesophageal Reflux, Peri-Rectal Swelling, Cholecystitis, Diverticulitis, Pancreatic Cancer, Emesis-Severe, Hepatomegaly, Hematemesis, Abrasion-Complicated, Rectal Urgency, Rectal Problems, Bowel Obstruction, Melena, Gi Bleeding, Cholelithiasis, Hematochezia, Rectal Bleeding-Major, Gastroparesis, Esophageal Reflux, Crohn's Disease, Bleeding Hemorrhoids, Rectal Bleeding, Gastric Cancer, Elevated Hepatic Enzymes, Bloating, Heartburn, Stool Color Change, Abdominal Pain, Gi Problem, Emesis, Constipation, Hiatal Hernia, Constipation, Gi Distress, Gi Pain, Gastric Cancer, Gout, Aspiration, Rectal Pain, Epigastric Pain, Cellulitis, Constipation, Gi Distress, Gi Pain, Epigastric Pain, Gas, Epigastric Pain, H. Pylori, Abdominal Pain Re-Evaluation, Gastrointestinal Tube Problem, Ulcerative Colitis, Peri-Rectal Abscess, Fecal Impaction, Hematemesis, Rectal Bleeding-Major, Abdominal Pain Pregnant, Diarrhea, Rectal Bleeding,

© 2025 Wong AH et al. *JAMA Network Open*.

Diarrhea, Rectal Bleeding-Minor, Rectal Bleeding, Anal Itching, Peri-Rectal Pain, Rectal Bleeding-Major, Diverticulitis, Diarrhea-Pediatric, Encopresis, Anal Fissure, Disphagia, Irritable Bowel Syndrome, Diverticulosis, Acute Renal Failure, Perineal Abscess, Hemorrhoids, Aphagia, Foreign Body In Rectum, Anal Irritation, Vomiting Blood, Cirrhosis, Jaw Swelling, Rectal Cancer, Inguinal Hernia, Perineal Pain, Hepatic Disease, Steatorrhea, Lactose Intolerance, Change In Bowel Habit, Peptic Ulcer Disease

Cardiovascular Conditions
Ventricular Tachycardia, Tachycardia, Pulmonary Embolism, Endocarditis, Tachycardia, Aortic Aneurysm, Hypotension, Heart Murmur, Tachycardia, Ventricular Tachycardia, Thoracic Aneurysm, Stroke Alert, Slow Hear Rate, Hereditary Hemorrhagic Telangiectasia, Circulatory Problem, Supraventricular Tachycardia (Svt), Heart Transplant Follow-Up, Heart Racing, Supraventricular Tachycardia (Svt), Heart Block, Congestive Heart Failure, Left Bundle Branch Block, Long Qt Syndrome, Rapid Heart Rate, Chest Pain, Irregular Heart Beat, Atrial Flutter, Palpitations, Bradycardia, Aicd Problem, Vascular Access Problem, Cardiac Arrest, Slow Hear Rate, Stemi Alert, Pacemaker Problem, Hypotension, Tachycardia, Abnormal Ecg, Stress Test, Cardiac Arrest, Atrial Fibrillation, Slow Hear Rate, Stemi Alert, Pacemaker Problem, Hypotension, Abnormal Ecg, Right Bundle Branch Block, Claudication, Cardiac Device Problem, Rapid Heart Rate, Brugada Syndrome, Shock From Defibrillator, Pacemaker Check, Chest Pain-Pediatric, Syncope, Shock From Defibrillator, Stent Removal, Respiratory Arrest, Hypercoagulable State, Cerebrovascular Accident, Slow Hear Rate, Hypertension, Deep Vein Thrombosis, Chest Tightness, Transient Ischemic Attack, Aicd Firing, Abnormal Chest X-Ray, Rapid Heart Rate, Heart Problem, Peripheral Artery Disease, Slow Heart Beat, Descending Aneurysm, Cardiomyopathy, Heart Failure Clinic, Echo

Immunological/Endocrine/Metabolic
Allergies, Angioedema, Tetanus Vaccine, Immunizations, Immunizations, Hypothermia, Bleeding/Bruising, Hypocalcemia, Allergic Reaction, Lupus, Immunosuppression, Bone Marrow Transplant, Allergic Rhinitis, Hematopoietic Stem Cell Transplant, Fibromyalgia, Hyperthermia, Lymphedema Garment Fitting, Allergic Rhinitis, Immunizations, Allergic Reaction-Major, Urticaria, Sickle Cell Anemia, Cerumen Impaction, Hypocalcemia, Goiter, Insulin Reaction, Hypothyroidism, Hypothermia, Hypospadias, Decreased Blood Sugar-No Symptoms, Nephrotic Syndrome, Polydipsia, Hyponatremia, Hot Flashes, Splenomegaly, Vitamin B Deficiency, Thyroid Problem, Hyperglycemia, Diabetes Mellitus 1, Hypercalcemia, Pancytopenia, Hypoglycemia, Hyperglycemia, Immunization, Diabetic Follow Up, Hypoglycemia, Renal Failure, Hyperthyroidism, Hyperglycemia, Diabetes Mellitus, Hyperthermia, High Potassium, Diabetes Mellitus 1, Glucose Check, Low Potassium, Vitamin B Deficiency, Adrenal Tumor, Obesity, Hyperlipidemia, Blood Sugar Problem, Hyperthermia, Thyroid Problem, Hypercalcemia, Low Potassium, High Potassium, Gout, Hypercalcemia, Hyperglycemia, Hyperglycemia, Diabetes, Chronic Kidney Disease, Chronic Renal Failure, Hypersensitivity Reaction, Sarcoidosis, Thyroid Nodule, Gestational Diabetes, Hyperparathyroidism, Diabetic Ketoacidosis, Juvenile Idiopathic Arthritis

Musculoskeletal Conditions
Rheumatoid Arthritis, Hip Pain, Bursitis, Extremity Weakness, Arm Swelling, Bone Pain, Leg Pain, Bunions, Spasms, Muscular Dystrophy, Hip Swelling, Shoulder Pain, Hip/Pelvis Pain, Scoliosis, Osteoporosis, Rib Pain, Wrist Swelling, Joint Pain, Muscle Pain, Carpal Tunnel, Temporomandibular Joint Pain, Craniofacial Abnormality, Osteoarthritis, Carpal Tunnel, Muscle Wasting, Finger Swelling, Muscle Pain, Back Problem, Elbow Pain, Bursitis, Carpal Tunnel, Knee Injury, Sternum Injury, Back Problem, Craniofacial Abnormality, Osteoarthritis, Thumb Injury, Tendonitis, Trigger Finger, Temporomandibular Joint Pain, Lymphedema Garment Fitting, Knee Problem, Knee Pain, Joint Swelling, Muscle Pain, Neck Pain, Back Pain, Foot Pain, Leg Swelling, Hand Swelling, Hip Pain, Hydrocele/ Hernia, Hernia, Knee Pain, Knee Problem, Osteoarthritis, Hammer Toe, Ganglion Cyst, Temporomandibular Joint Pain, Hand Swelling, Back Swelling, Hip Pain, Lower Back Pain, Neck Pain, Heel Pain, Back Pain, Buttocks Pain, Groin Pain, Sternum Swelling, Elbow Problem, Buttocks Pain, Gait Problem, Muscle Wasting, Upper Back Pain, Arm Problem, Hand Problem, Arm Pain,

© 2025 Wong AH et al. *JAMA Network Open*.

Osteomyelitis, Sciatica, Finger Pain, Thumb Swelling, Ankle Pain, Jaw Pain, Wrist Pain, Ankle Pain, Thumb Pain, Inguinal Hernia

Respiratory Conditions

Dyspnea-12 Years Or Less, Snoring, Pleurisy, Pneumonia, Breathing Difficulty-12 Years Or Less, Cold Exposure, Tracheostomy Tube Evaluation, Dyspnea-12 Years Or Less, Shortness Of Breath, Shortness Of Breath-Pediatric, Asphyxia, Croup, Apnea, Respiratory Difficulties, Croup With Respiratory Distress, Bronchitis, Wheezing, Respiratory Distress-Pediatric, Stridor, Copd, Breathing Problem, Asthma, Lung Nodule(S), Croup, Cough, Nasal Swelling, Cough, Bronchitis, Flu Like Symptoms, Breathing Problem, Respiratory Distress, Breathing Difficulty, Copd, Dyspnea, Hemoptysis, Wheezing- Peds Needing Stat Intervention, Respiratory Distress, Inhalation Injury, Inhalation Injury- Severe Dyspnea, Wheezing- Adult Needing Stat Intervention, Respiratory Arrest, Snoring, Airway Obstruction, Lung Cancer, Toxic Inhalation, Aspiration, Airway Obstruction, Shortness Of Breath, Shortness Of Breath-Pediatric, Dyspnea-12 Years Or Less, Pleurisy, Shortness Of Breath, Cough, Asthma, Hemoptysis- Major, Laryngitis, Bipap, Recurrent Pneumonia, Asthma-Pediatric, Interstitial Lung Disease, Exercise Induced Shortness Of Breath

Miscellaneous

Morning After Pill, Chemical Exposure, Ekg, Extremity Pain, Other, Seroma, Insect Bite- Marked Reaction, Congenital Central Hypoventilation Syndrome, Stuttering, Near Drowning, Care Planning, Subungual Hematoma, Tetanus Vaccine, Ekg, Pallor, Letter For School/Work, Mouth Pain, Dry Mouth, Cold Feet, Tearing, Eye, Sexual Encounter Unplanned, Triage, Confirmation, Fussy, Patient Education, Other, Sleeping Problem, Elective Abortion, Dense Breasts, Dense Breasts, Palpable Lymph Node, Pallor, Fyi, Jail Clearance, Edema, Rapid Heart Rate, Decompression Illness, Alleged Sexual Assault, Thrush, Concussion, Fever-9 Weeks To 74 Years, Edema-12 Years Or Less, Srp&Amp, Edema-12 Years Or Less, Second Opinion, Weight Gain, Abnormal Lung Scan, Synocope, Drug / Alcohol Assessment, Foreign Body Sensation, Frostbite, Coagulation Disorder, Rupture Of Membranes, Personal Problem, Work Exposure, Continuum Of Care, Irregular Eye Movement, Procedure, Miscellaneous, Results, Miscellaneous, Confirmation, Environmental Exposure, Environmental Exposure, Covid-19 Result, Secretions, Poisoning, Hypertension- Pregnant, Carbon Monoxide (Co) Exposure, Social Work, Safety Eval / Driving, Advice Only, Mobility, Triage Medical Complaint, Work Exposure, Gingivitis, Environmental Exposure, Flank Pain, Fatigue, Lethargy, Excessive Daytime Sleepiness, Snoring, Neutropenia, Extremity Weakness, Dehydration, Nausea, Abnormal Imaging, Sickle Cell Pain Crisis, Edema-12 Years Or Less, Blepharitis, Cold Symptoms, Dizziness, Flu Like Symptoms, Pruritus, Difficulty Swallowing, Poor Circulation, Illness, Extremity Pain, Swelling At Surgical Site, Fever Immunocompromised, Headache- Recurrent Or Known Dx Migraines, Pain With Swallowing, Dehydration, Lethargy, Neck Stiffness, Shock From Defibrillator, Tongue Swelling, Difficulty Maintaining Sleep, Difficulty Initiating Sleep, Excessive Daytime Sleepiness, Crying, Blood Pressure Check, Cold Extremity, Fussy, Confirmation, Urgent Visit, Headache, Headache Re-Evaluation, Musculoskeletal Pain, Headache Infusion Request, Loss Of Appetite, Numbness, Palpitations-Adolescent 12 To 17 Years, Initial Evaluation, Weakness, Sore, Hand Pain, Post Void Dribbling, Weight Gain, Headache Re-Evaluation, Difficulty Walking, Rapid Heart Rate, Edema-12 Years Or Less, Cyst, Heel Spurs, Vitamin B Deficiency, Lip Irritation, Tongue Swelling- Major, Side Pain, Weight Loss, Pain, Pain With Inspiration, Sleep Disturbance, Night Sweats, Burning Eyes, Malaise, Lip Swelling, Slow Hear Rate, Tremors, Tingling, Irregular Eye Movement, Unresponsive, Night Sweats, Malaise, Nasal Swelling, Lip Swelling, Slow Hear Rate, Loss Of Vision, Tremors, Tingling, Irregular Eye Movement, Unresponsive, Excessive Sleepiness/Hypersomnia, Fever-75 Years Or Older, Anemia, Chills, Generalized Body Aches, Excessive Sweating, Decreased Blood Sugar-Symptomatic, Oral Pain, Dyspnea, Toe Swelling, Hiccups, Loss Of Consciousness, Itch, Drooling, Elevated Blood Sugar-Symptomatic, Dehydration-24 Months Or Less, Hyperventilating, Lower Extremity Pain, Suprapubic Pain, Chest Swelling, Axilla Abscess, General Physical, Choking, Failure To Thrive, Cold Like Symptoms, Shaking, Glucose Check, Mobility, Restless Leg Syndrome, Near Syncope, Paresthesia, Callouses, Safety Eval / Driving, Sickle Cell Pain, Nocturnal Enuresis, Mouth Pain, Facial Swelling, Malaise, Pallor, Periorbital Swelling, Platelets Reduction, Red Blood Cell Exchange, Thrombocytopenia, Pancytopenia, Leukocytes Reduction, Leukocytes Reduction,

Hoarseness, Facial Pain, Tinnitus, Toe Pain, Tailbone Pain, Chest Pain/Discomfort, Mass, Nocturia, Pain, Post Nasal Drip, Sleep Apnea, Decreased Fetal Movement, Rapid Heart Rate, Arm Problem, Hand Problem, Arm Pain, Heat Exposure, Abscess, Hoarseness, Decreased Blood Sugar-No Symptoms, Uri, Housing, Social Work, Safety Eval / Driving, Mobility, Advice Only, Galactorrhea, Gingivitis, Personal Problem, Alleged Sexual Assault, Rodent Bite, Foreign Body Sensation, Second Opinion, Discharged, Coagulation Disorder, Bunions, Sunburn, Weight Gain, Frostbite, Work Exposure, Continuum Of Care, Environmental Exposure, Admission, Therapeutic Plasma Exchange, Eyelid Pain, Homeless, Leg Pains At Night, Slurred Speech, Sciatica, Foot Swelling, Color Reduction, Throat Pain, Migraine, Cyanosis, Speech Problem, Vomiting, Sore Throat, Decreased Oxygen Level-No Symptoms, Tics, Cyanosis, Oliguria, Ankle Pain, Decreased Level Of Consciousness, Arthritis, Decreased Level Of Consciousness, Ankle Pain, Neck Swelling, Nasal Polyps, Earache, Acute Diaphoresis, Abnormal Coagulation Labs On Screening, Impacted Tooth, Thumb Pain, Elevated Psa, Well Child, Referral, P, Ingestion, Antibiotic Infusion, Ant Bite, Insomnia, Animal Bite, Eating Disorder, Ingestion, Family Problem, Abdominal Distention, Nasal Pain, Bat Bite Or Exposure, Color Reduction, Foreign Body, Pepper Spray, Detox Evaluation, Ear Pain, Breast Pain, Annual Wellness Exam, Employment Physical, Foot Problem, Radiation Exposure, Otalgia, Extraction, Marine Bite Or Sting, Mouth Injury, Febrile Seizure, Wrist Pain, Headache- New Onset Or New Symptoms, Abnormal Lab, Chronic Pain, Dental Pain, Nose Problem, Contraception, Drug Screen, Dental Problem, Dental Swelling, Caries, Dental Injury, Developmental Delay, Sternum Pain, Mouth Swelling, Elevated Blood Sugar-No Symptoms, Mouth Lesions, Incisional Pain, Oral Swelling, Gout Pain, Newborn Eval After Pre Ed Or Ed Delivery, Assault Victim, Vomiting Blood, Toxidrome, Abnormal Ultrasound, Feeding Intolerance, Medical Problem- Major, Wellness Visit, Pre-Certification, Nutrition Counseling, Post T And A Bleed, Epistaxis, Electric Exposure/Shock-Asymptomatic, Biological Exposure, Trauma, Wound Dehiscence, Platelets, Radiation, Breast Problem, Rejection (Transplant Rejection), Covid-19 Vaccine Side Effects, Outreach Worker Note, Child Life, Conservatorship, Bmt Family Meeting, Organophosphate Poisoning, Mcm_Service Plan – Update, Mcm_Service Plan Â€“ Update, Prep (Pre-Exposure Prophylaxis), Treatment Question, Hyperopia, Rejection (Transplant Rejection), Rejection, Prep

Neurological Conditions

Facial Droop, Nystagmus, Eye Twitching, Seizure- Pregnant, Narcolepsy, Excessive Sleepiness/Hypersomnia, Restless Sleep, Memory Loss, Unresponsive, Seizures, Altered Mental Status, Facial Numbness, Vertigo- Recurrent, Multiple Sclerosis, Myasthenia Gravis, Dystonic Reaction, Peripheral Neuropathy, Focal Motor Weakness, Nystagmus, Paresthesia, Neuropsych Testing, Hypoxic Ischemic Encephalopathy, Multiple Sclerosis, Seizure Re-Evaluation, Facial Droop, Paralysis, Seizure- New Onset, Seizure- Prior Hx Of, Seizure- Actively Seizing On Arrival, Aphasia, Adhd, Migraine, Neurologic Problem, Autism Evaluation, Pseudotumor Cerebri, Neuroma, Sleepwalking, ALS (Amyotrophic Lateral Sclerosis), Tourette Syndrome, ALS

Unknown Conditions

Unknown, Other

© 2025 Wong AH et al. *JAMA Network Open*.

eTable 2. A list of All Included Features and Associated Descriptions. All features are binary unless otherwise specified.

Feature	Description	Parameters
**Demographic Information**		
age_continuous	Age in years	Continuous<br><br>Mean (SD): 46.28 (20.04)<br>Median: 46<br>Min: 18<br>Max: 122<br><br>*Preprocessing*:<br>Capped at 110 to avoid skewness
WEIGHT	Weight in kg (kilograms)	Continuous<br><br>Mean (SD): 82.66 (21.27)<br>Median: 79.8<br>Min: 34.4<br>Max: 204
TOBACCO_PAK_PER_DY	Use of tobacco (Packs per day)	Continuous<br><br>Mean (SD): 0.56 (1.42)<br>Median: 0.5<br>Min: 0<br>Max: 400
**Patient History**		
ED_visits	Number of ED visits (in total)	Continuous<br><br>Mean (SD): 2.77 (7.12)<br>Median: 1<br>Min: 0<br>Max: 130
Number.of.ED.visits.in.past.year	Number of ED visits (in past year)	Continuous<br><br>Mean (SD): 3.78 (7.28)<br>Median: 2<br>Min: 1<br>Max: 417

© 2025 Wong AH et al. *JAMA Network Open*.

FALL.RISK.TOTAL.SCORE	Numerical score or assessment used to evaluate an individual's risk of experiencing a fall	Categorical<br><br>0<br>1<br>2<br>3<br>4<br>5
Num_hospital_admission	Number of hospital admissions (in past year)	Continuous<br><br>Mean (SD): 30.81 (45.04)<br>Median: 15<br>Min: 0<br>Max: 983
BMI	Body mass index	Continuous<br><br>Mean (SD): 28 (3.92)<br>Median: 27.66<br>Min: 9.73<br>Max: 92.59
previous_restraint_cen	Presence of previous restraints (y/n)	0<br>1
previous_restraint_cen_num	Number of times restrained previously (violent physical restraint and chemical sedation)	Continuous<br><br>Mean (SD): 0.09 (0.67)<br>Median: 0<br>Min: 0<br>Max: 112
Financial_Class_BCBS	Insurance	0<br>1
Financial_Class_Commercial		0<br>1
Financial_Class_Managed Care		0<br>1
Financial_Class_Medicaid		0<br>1
Financial_Class_Medicaid Managed Care		0<br>1
Financial_Class_Medicare		0<br>1

© 2025 Wong AH et al. *JAMA Network Open*.

Financial_Class_Medicare Managed Care		0<br>1
Financial_Class_Other		0<br>1
Financial_Class_Self-Pay		0<br>1
Financial_Class_Special Programs		0<br>1
Financial_Class_Tricare		0<br>1
Financial_Class_Worker's Comp		0<br>1
EMPLOYMENT.STATUS_Disabled	Employment status	0<br>1
EMPLOYMENT.STATUS_Full Time		0<br>1
EMPLOYMENT.STATUS_Not Employed		0<br>1
EMPLOYMENT.STATUS_On Active Military Duty		0<br>1
EMPLOYMENT.STATUS_Part Time		0<br>1
EMPLOYMENT.STATUS_Retired		0<br>1
EMPLOYMENT.STATUS_Self Employed		0<br>1
EMPLOYMENT.STATUS_Student - Full Time		0<br>1
EMPLOYMENT.STATUS_Student - Part Time		0<br>1
EMPLOYMENT.STATUS_Unknown		0<br>1
INTRPTR_NEEDED_YN_Y	Whether an interpreter is needed to assist with questions	0<br>1
GENDER_Female	Gender of patient	0<br>1
GENDER_Male		0<br>1
GENDER_Unknown		0

© 2025 Wong AH et al. *JAMA Network Open*.

		1
LANGUAGE_English	Primary language	0<br>1
LANGUAGE_Other/Unknown		0<br>1
LANGUAGE_Sign Language		0<br>1
LANGUAGE_Spanish		0<br>1
HOMELESS_YN_No	Whether an individual is homeless	0<br>1
HOMELESS_YN_Yes		0<br>1
SMOKING_STATUS_Current	Smoking status	0<br>1
SMOKING_STATUS_Former		0<br>1
SMOKING_STATUS_Never		0<br>1
SMOKING_STATUS_Unknown		0<br>1
DRUG.USER_Former	Drug use behavior	0<br>1
DRUG.USER_No		0<br>1
DRUG.USER_Unknown		0<br>1
DRUG.USER_Yes		0<br>1
Cannabis	Drug Type – indicates whether the patient has a history of drug use<br><br>Drug Type: Cannabis - Includes Marijuana, cannabis, hashish, synthetic cannabinoids (e.g., K2, Spice)<br><br>Drug Type: Depressants - This includes Benzodiazepines (e.g., alprazolam, diazepam, clonazepam, lorazepam,	0<br>1
Depressants		0<br>1

© 2025 Wong AH et al. *JAMA Network Open*.

Hallucinogens	chlordiazepoxide), Barbiturates (e.g., phenobarbital), Flunitrazepam, Alprazolam, Diazepam, Clonazepam, Phenobarbital, Chlordiazepoxide, and Lorazepam.<br><br>Drug Type: Hallucinogens - This includes LSD, Psilocybin, DMT, Ketamine, Mescaline, and Salvia.<br><br>Drug Type: Opioids - This includes Oxycodone, Heroin, Methadone, Fentanyl, Morphine, Hydrocodone, Hydromorphone, Opium, Percocet, and Codeine.<br><br>Drug Type: Other - Substances that do not fall under the major categories above. This includes Amyl nitrate, Anabolic steroids, Solvent inhalants, Woolly/Illy, Speedball, Other - see comments, Synthetic cathinones (bath salts), IV drug use, "Crack" cocaine, PCP, GHB, Nitrous oxide, Unknown (if explicitly recorded as "Unknown" but not coded under the unknown category), BUP (Buprenorphine), Kratom, and Methaqualone.<br><br>Drug Type: Stimulants - This includes Cocaine, Amphetamines, Methamphetamines, MDMA (Ecstasy), Methylphenidate, and Crystal Meth.<br><br>Drug Type: Unknown - indicates that the patient's substance use history is marked as unknown.	0<br>1
Opioids		0<br>1
Other		0<br>1
Stimulants		0<br>1
Unknown		0<br>1
EMPLOYMENT_Active duty	Employment status	0<br>1
EMPLOYMENT_Disabled		0<br>1
EMPLOYMENT_Employed, full time		0<br>1
EMPLOYMENT_Employed, part time		0<br>1
EMPLOYMENT_Retired		0<br>1
EMPLOYMENT_Student		0<br>1
EMPLOYMENT_Unemployed		0<br>1

© 2025 Wong AH et al. *JAMA Network Open*.

EMPLOYMENT_Unknown		0<br>1
ALCOHOL_No	Presence of Alcohol Use<br><br>Whether the patient has reported alcohol use	0<br>1
ALCOHOL_Unknown		0<br>1
ALCOHOL_Yes		0<br>1
accomodations.cat_No	Whether a patient has specific accommodations (needs)	0<br>1
accomodations.cat_Yes		0<br>1
**Initial Vital Signs (on ED arrival)**		
SYSTOLIC_bp	Blood pressure - systolic	Continuous<br><br>Mean (SD): 138.34 (24.12)<br>Median: 135<br>Min: 69<br>Max: 232<br><br>*Preprocessing*:<br>Trimmed on 1st and 99th percentiles
DIASTOLIC_bp	Blood pressure - diastolic	Continuous<br><br>Mean (SD): 80.23 (13.74)<br>Median: 80<br>Min: 32<br>Max: 141<br><br>*Preprocessing:*<br>Trimmed on 1st and 99th percentiles
PULSE	Pulse	Continuous<br><br>Mean (SD): 86.53 (17.35)<br>Median: 85<br>Min: 39<br>Max: 196
TEMPERATURE	Temperature (in Fahrenheit)	Continuous

© 2025 Wong AH et al. *JAMA Network Open*.

		Mean (SD): 97.96 (0.92)<br>Median: 98<br>Min: 93.7<br>Max: 104.2
SPO2	Oxygen saturation (in percentage)	Continuous<br><br>Mean (SD): 97.76 (2.36)<br>Median: 98<br>Min: 70<br>Max: 100
RESP	Respiratory rate	Continuous<br><br>Mean (SD): 18.87 (3.14)<br>Median: 18<br>Min: 11<br>Max: 71<br><br>*Preprocessing*:<br>Trimmed on 1st and 99th percentiles
**Visit Characteristics**		
ESCORTED.BY_Co-Worker	Who accompanied the patient upon arrival at healthcare facility	0<br>1
ESCORTED.BY_Family member		0<br>1
ESCORTED.BY_Friend		0<br>1
ESCORTED.BY_Other		0<br>1
ESCORTED.BY_Self		0<br>1
ESCORTED.BY_Spouse		0<br>1
ESCORTED.BY_Unknown		0<br>1
PSYCHIATRIST.ON.TREATMENT_TEAM_NO	Presence of psychiatrist on treatment team	0<br>1
PSYCHIATRIST.ON.TREATMENT_TEAM_YES		0<br>1

© 2025 Wong AH et al. *JAMA Network Open*.

<table>
<tr><td>ESI_LEVEL_1</td><td rowspan="6">ESI (Emergency Severity Index) is a tool used for triage to assess the severity of a patient's condition upon arrival at the emergency department. ESI is a five-level acuity scale that helps emergency healthcare providers prioritize patients based on the severity of their illness or injury.

ESI Level 1 (Immediate): Patients in this category are in critical condition and require immediate medical attention. They may have life-threatening injuries or illnesses that need rapid intervention.

ESI Level 2 (Emergent): Patients in this category have a high level of urgency. They may have a severe medical condition or injury that needs prompt attention.

ESI Level 3 (Urgent): Patients in this category require evaluation and care relatively quickly, but their conditions are not immediately life-threatening.

ESI Level 4 (Less Urgent): Patients in this category have non-severe conditions that do not require immediate attention. They can wait for a longer period without risking their health.

ESI Level 5 (Non-urgent): Patients in this category have minor illnesses or injuries that are not time-sensitive. They can wait longer for care without significant risk.</td><td>0
1</td></tr>
<tr><td>ESI_LEVEL_2</td><td>0
1</td></tr>
<tr><td>ESI_LEVEL_3</td><td>0
1</td></tr>
<tr><td>ESI_LEVEL_4</td><td>0
1</td></tr>
<tr><td>ESI_LEVEL_5</td><td>0
1</td></tr>
<tr><td>ESI_LEVEL_Unknown</td><td>0
1</td></tr>
<tr><td colspan="3">Chief Complaint</td></tr>
<tr><td>ONC_CC</td><td>Chief Complaint: Oncological conditions related to cancer and oncological diseases.</td><td>0
1</td></tr>
<tr><td>DRG_CC</td><td>Chief Complaint: Substance Use Disorder; Drug and alcohol-related conditions associated with substance use, dependence, withdrawal, and overdose.</td><td>0
1</td></tr>
<tr><td>ENT_CC</td><td>Chief Complaint: ENT (ear, nose throat) conditions, including both infectious and structural disorders.</td><td>0
1</td></tr>
<tr><td>OPT_CC</td><td>Chief Complaint: Ophthalmic conditions affecting the eyes and vision.</td><td>0
1</td></tr>
</table>

© 2025 Wong AH et al. *JAMA Network Open*.

URC_CC	Chief Complaint: Urological and Reproductive conditions; Urological and reproductive conditions, as well as pregnancy related issues and sexually transmitted diseases.	0<br>1
MEN_CC	Chief Complaint: Psychiatric; Mental health conditions related to psychiatric and behavioral health.	0<br>1
AGIT_CC	Chief Complaint: Agitation related conditions	0<br>1
INJ_CC	Chief Complaint: Injury-related conditions	0<br>1
INF_CC	Chief Complaint: Infectious Diseases; Infectious diseases including bacterial, viral, fungal, and parasitic infections.	0<br>1
DERM_CC	Chief Complaint: Dermatological conditions	0<br>1
PRO_CC	Chief Complaint: Procedural; Procedures and follow-ups-medical, surgical, and post-operative procedures as well as follow-ups for previous treatments and interventions.	0<br>1
GI_CC	Chief Complaint: Gastrointestinal	0<br>1
CAR_CC	Chief Complaint: Cardiovascular	0<br>1
IMM_CC	Chief Complaint: Immunological/endocrine/metabolic conditions	0<br>1
MSK_CC	Chief Complaint: Musculoskeletal	0<br>1
RESP_CC	Chief Complaint: Respiratory	0<br>1
MISC_CC	Chief Complaint: Miscellaneous; Miscellaneous conditions that do not fit into other categories, such as unspecified symptoms, environmental exposures, routine evaluations, and non-specific medical concerns.	0<br>1
NEURO_CC	Chief Complaint: Neurological	0<br>1
UNK_CC	Chief Complaint: Unknown (Unspecified)	0<br>1
**Past Medical History categories (CCSR groupings - https://hcup-us.ahrq.gov/toolssoftware/ccsr/ccs_refined.jsp)**		
pmh_INF004	Past Medical History: Fungal infections	No<br>Yes
pmh_INF009	Past Medical History: Parasitic, other specified and unspecified infections	No<br>Yes

© 2025 Wong AH et al. *JAMA Network Open*.

pmh_INF001	Past Medical History: Tuberculosis	No<br>Yes
pmh_NEO015	Past Medical History: Gastrointestinal cancers - colorectal	No<br>Yes
pmh_NEO051	Past Medical History: Endocrine system cancers - pancreas	No<br>Yes
pmh_NEO022	Past Medical History: Respiratory cancers	No<br>Yes
pmh_NEO024	Past Medical History: Sarcoma	No<br>Yes
pmh_NEO025	Past Medical History: Skin cancers - melanoma	No<br>Yes
pmh_NEO026	Past Medical History: Skin cancers - basal cell carcinoma	No<br>Yes
pmh_NEO028	Past Medical History: Skin cancers - all other types	No<br>Yes
pmh_NEO030	Past Medical History: Breast cancer - all other types	No<br>Yes
pmh_NEO031	Past Medical History: Female reproductive system cancers - uterus	No<br>Yes
pmh_NEO042	Past Medical History: Male reproductive system cancers - all other types	No<br>Yes
pmh_NEO043	Past Medical History: Urinary system cancers - bladder	No<br>Yes
pmh_NEO047	Past Medical History: Urinary system cancers - all other types	No<br>Yes
pmh_NEO048	Past Medical History: Nervous system cancers - brain	No<br>Yes
pmh_NEO049	Past Medical History: Nervous system cancers - all other types	No<br>Yes
pmh_RSP002	Past Medical History: Pneumonia (except that caused by tuberculosis)	No<br>Yes
pmh_NEO070	Past Medical History: Secondary malignancies	No<br>Yes
pmh_NEO071	Past Medical History: Malignant neoplasm, unspecified	No<br>Yes
pmh_NEO058	Past Medical History: Non-Hodgkin lymphoma	No<br>Yes

© 2025 Wong AH et al. *JAMA Network Open*.

pmh_SKN001	Past Medical History: Skin and subcutaneous tissue infections	No<br>Yes
pmh_NEO061	Past Medical History: Leukemia - chronic lymphocytic leukemia (CLL)	No<br>Yes
pmh_NEO064	Past Medical History: Leukemia - all other types	No<br>Yes
pmh_NEO066	Past Medical History: Malignant neuroendocrine tumors	No<br>Yes
pmh_NEO073	Past Medical History: Benign neoplasms	No<br>Yes
pmh_CIR019	Past Medical History: Heart failure	No<br>Yes
pmh_INF003	Past Medical History: Bacterial infections	No<br>Yes
pmh_NEO072	Past Medical History: Neoplasms of unspecified nature or uncertain behavior	No<br>Yes
pmh_END001	Past Medical History: Thyroid disorders	No<br>Yes
pmh_END003	Past Medical History: Diabetes mellitus with complication	No<br>Yes
pmh_END002	Past Medical History: Diabetes mellitus without complication	No<br>Yes
pmh_END015	Past Medical History: Other specified and unspecified endocrine disorders	No<br>Yes
pmh_END013	Past Medical History: Pituitary disorders	No<br>Yes
pmh_END016	Past Medical History: Other specified and unspecified nutritional and metabolic disorders	No<br>Yes
pmh_INF002	Past Medical History: Septicemia	No<br>Yes
pmh_END010	Past Medical History: Disorders of lipid metabolism	No<br>Yes
pmh_MUS033	Past Medical History: gout	No<br>Yes
pmh_END011	Past Medical History: Fluid and electrolyte disorders	No<br>Yes
pmh_CIR018	Past Medical History: Cardiac arrest and ventricular fibrillation	No<br>Yes

© 2025 Wong AH et al. *JAMA Network Open*.

pmh_BLD002	Past Medical History: Hemolytic anemia	No<br>Yes
pmh_BLD005	Past Medical History: Sickle cell trait/anemia	No<br>Yes
pmh_BLD004	Past Medical History: Acute posthemorrhagic anemia	No<br>Yes
pmh_BLD003	Past Medical History: Aplastic anemia	No<br>Yes
pmh_BLD006	Past Medical History: Coagulation and hemorrhagic disorders	No<br>Yes
pmh_BLD010	Past Medical History: Other specified and unspecified hematologic conditions	No<br>Yes
pmh_NVS011	Past Medical History: Neurocognitive disorders	No<br>Yes
pmh_MBD025	Past Medical History: Other specified substance-related disorders	No<br>Yes
pmh_MBD013	Past Medical History: Miscellaneous mental and behavioral disorders/conditions	No<br>Yes
pmh_MBD001	Past Medical History: Schizophrenia spectrum and other psychotic disorders	No<br>Yes
pmh_MBD005	Past Medical History: Anxiety and fear-related disorders	No<br>Yes
pmh_MBD009	Past Medical History: Personality disorders	No<br>Yes
pmh_MBD020	Past Medical History: Sedative-related disorders	No<br>Yes
pmh_MBD019	Past Medical History: Cannabis-related disorders	No<br>Yes
pmh_MBD021	Past Medical History: Stimulant-related disorders	No<br>Yes
pmh_MBD023	Past Medical History: Inhalant-related disorders	No<br>Yes
pmh_MAL010	Past Medical History: Other specified and unspecified congenital anomalies	No<br>Yes
pmh_NVS016	Past Medical History: Sleep wake disorders	No<br>Yes
pmh_MBD010	Past Medical History: Feeding and eating disorders	No<br>Yes

© 2025 Wong AH et al. *JAMA Network Open*.

pmh_SYM008	Past Medical History: Symptoms of mental and substance use conditions	No<br>Yes
pmh_MBD007	Past Medical History: Trauma- and stressor-related disorders	No<br>Yes
pmh_MBD014	Past Medical History: Neurodevelopmental disorders	No<br>Yes
pmh_EAR004	Past Medical History: Hearing loss	No<br>Yes
pmh_NVS006	Past Medical History: Other nervous system disorders (often hereditary or degenerative)	No<br>Yes
pmh_INJ030	Past Medical History: Drug induced or toxic related condition	No<br>Yes
pmh_NVS010	Past Medical History: Headache; including migraine	No<br>Yes
pmh_NVS007	Past Medical History: Cerebral palsy	No<br>Yes
pmh_NVS008	Past Medical History: Paralysis (other than cerebral palsy)	No<br>Yes
pmh_NVS009	Past Medical History: Epilepsy; convulsions	No<br>Yes
pmh_NVS020	Past Medical History: Other nervous system disorders (neither hereditary nor degenerative)	No<br>Yes
pmh_NVS017	Past Medical History: Nerve and nerve root disorders	No<br>Yes
pmh_NVS015	Past Medical History: Polyneuropathies	No<br>Yes
pmh_NVS018	Past Medical History: Myopathies	No<br>Yes
pmh_EYE005	Past Medical History: Retinal and vitreous conditions	No<br>Yes
pmh_EYE003	Past Medical History: Glaucoma	No<br>Yes
pmh_EAR006	Past Medical History: Other specified and unspecified disorders of the ear	No<br>Yes
pmh_EYE002	Past Medical History: Cataract and other lens disorders	No<br>Yes
pmh_EYE010	Past Medical History: Blindness and vision defects	No<br>Yes

© 2025 Wong AH et al. *JAMA Network Open*.

pmh_EYE001	Past Medical History: Cornea and external disease	No<br>Yes
pmh_EYE008	Past Medical History: Oculofacial plastics and orbital conditions	No<br>Yes
pmh_EYE006	Past Medical History: Neuro-ophthalmology	No<br>Yes
pmh_EAR002	Past Medical History: Diseases of middle ear and mastoid (except otitis media)	No<br>Yes
pmh_CIR002	Past Medical History: Acute rheumatic heart disease	No<br>Yes
pmh_CIR001	Past Medical History: Chronic rheumatic heart disease	No<br>Yes
pmh_CIR007	Past Medical History: Essential hypertension	No<br>Yes
pmh_CIR011	Past Medical History: Coronary atherosclerosis and other heart disease	No<br>Yes
pmh_INF006	Past Medical History: HIV infection	No<br>Yes
pmh_CIR004	Past Medical History: Endocarditis and endocardial disease	No<br>Yes
pmh_CIR006	Past Medical History: Pericarditis and pericardial disease	No<br>Yes
pmh_CIR003	Past Medical History: Nonrheumatic and unspecified valve disorders	No<br>Yes
pmh_CIR005	Past Medical History: Myocarditis and cardiomyopathy	No<br>Yes
pmh_CIR017	Past Medical History: Cardiac dysrhythmias	No<br>Yes
pmh_CIR021	Past Medical History: Acute hemorrhagic cerebrovascular disease	No<br>Yes
pmh_CIR023	Past Medical History: Occlusion or stenosis of precerebral or cerebral arteries without infarction	No<br>Yes
pmh_CIR024	Past Medical History: Other and ill-defined cerebrovascular disease	No<br>Yes
pmh_CIR025	Past Medical History: Sequela of cerebral infarction and other cerebrovascular disease	No<br>Yes
pmh_CIR026	Past Medical History: Peripheral and visceral vascular disease	No<br>Yes

© 2025 Wong AH et al. *JAMA Network Open*.

pmh_CIR029	Past Medical History: Aortic; peripheral; and visceral artery aneurysms	No<br>Yes
pmh_CIR032	Past Medical History: Other specified and unspecified circulatory disease	No<br>Yes
pmh_CIR033	Past Medical History: Acute phlebitis; thrombophlebitis and thromboembolism	No<br>Yes
pmh_CIR034	Past Medical History: Chronic phlebitis; thrombophlebitis and thromboembolism	No<br>Yes
pmh_DIG014	Past Medical History: Hemorrhoids	No<br>Yes
pmh_CIR039	Past Medical History: Other specified diseases of veins and lymphatics	No<br>Yes
pmh_CIR031	Past Medical History: Hypotension	No<br>Yes
pmh_RSP006	Past Medical History: Other specified upper respiratory infections	No<br>Yes
pmh_RSP007	Past Medical History: Other specified and unspecified upper respiratory disease	No<br>Yes
pmh_MUS006	Past Medical History: Osteoarthritis	No<br>Yes
pmh_RSP016	Past Medical History: Other specified and unspecified lower respiratory disease	No<br>Yes
pmh_EXT025	Past Medical History: External cause codes: complications of medical and surgical care, initial encounter	No<br>Yes
pmh_RSP003	Past Medical History: Influenza	No<br>Yes
pmh_RSP008	Past Medical History: Chronic obstructive pulmonary disease and bronchiectasis	No<br>Yes
pmh_RSP009	Past Medical History: Asthma	No<br>Yes
pmh_INF008	Past Medical History: Viral infection	No<br>Yes
pmh_RSP010	Past Medical History: Aspiration pneumonitis	No<br>Yes
pmh_RSP013	Past Medical History: Lung disease due to external agents	No<br>Yes
pmh_RSP012	Past Medical History: Respiratory failure; insufficiency; arrest	No<br>Yes

© 2025 Wong AH et al. *JAMA Network Open*.

pmh_MUS029	Past Medical History: Disorders of jaw	No<br>Yes
pmh_FAC022	Past Medical History: Acquired absence of limb or organ	No<br>Yes
pmh_DIG003	Past Medical History: Diseases of mouth; excluding dental	No<br>Yes
pmh_DIG004	Past Medical History: Esophageal disorders	No<br>Yes
pmh_DIG005	Past Medical History: Gastroduodenal ulcer	No<br>Yes
pmh_DIG007	Past Medical History: Gastritis and duodenitis	No<br>Yes
pmh_DIG008	Past Medical History: Other specified and unspecified disorders of stomach and duodenum	No<br>Yes
pmh_INF005	Past Medical History: Foodborne intoxications	No<br>Yes
pmh_DIG009	Past Medical History: Appendicitis and other appendiceal conditions	No<br>Yes
pmh_DIG010	Past Medical History: Abdominal hernia	No<br>Yes
pmh_DIG011	Past Medical History: Regional enteritis and ulcerative colitis	No<br>Yes
pmh_DIG012	Past Medical History: Intestinal obstruction and ileus	No<br>Yes
pmh_DIG013	Past Medical History: Diverticulosis and diverticulitis	No<br>Yes
pmh_DIG025	Past Medical History: Other specified and unspecified gastrointestinal disorders	No<br>Yes
pmh_DIG016	Past Medical History: Peritonitis and intra-abdominal abscess	No<br>Yes
pmh_DIG015	Past Medical History: Anal and rectal conditions	No<br>Yes
pmh_DIG019	Past Medical History: Other specified and unspecified liver disease	No<br>Yes
pmh_DIG018	Past Medical History: Hepatic failure	No<br>Yes
pmh_DIG017	Past Medical History: Biliary tract disease	No<br>Yes

© 2025 Wong AH et al. *JAMA Network Open*.

pmh_GEN007	Past Medical History: Other specified and unspecified diseases of bladder and urethra	No<br>Yes
pmh_DIG020	Past Medical History: Pancreatic disorders (excluding diabetes)	No<br>Yes
pmh_GEN001	Past Medical History: Nephritis; nephrosis; renal sclerosis	No<br>Yes
pmh_GEN002	Past Medical History: Acute and unspecified renal failure	No<br>Yes
pmh_GEN006	Past Medical History: Other specified and unspecified diseases of kidney and ureters	No<br>Yes
pmh_GEN005	Past Medical History: Calculus of urinary tract	No<br>Yes
pmh_GEN017	Past Medical History: Nonmalignant breast conditions	No<br>Yes
pmh_GEN018	Past Medical History: Inflammatory diseases of female pelvic organs	No<br>Yes
pmh_GEN019	Past Medical History: Endometriosis	No<br>Yes
pmh_GEN023	Past Medical History: Menopausal disorders	No<br>Yes
pmh_GEN024	Past Medical History: Female infertility	No<br>Yes
pmh_GEN025	Past Medical History: Other specified female genital disorders	No<br>Yes
pmh_PRG006	Past Medical History: Molar pregnancy and other abnormal products of conception	No<br>Yes
pmh_PRG004	Past Medical History: Induced abortion and complications of termination of pregnancy	No<br>Yes
pmh_PRG009	Past Medical History: Early, first or unspecified trimester hemorrhage	No<br>Yes
pmh_PRG020	Past Medical History: Hypertension and hypertensive-related conditions complicating pregnancy; childbirth; and the puerperium	No<br>Yes
pmh_PRG015	Past Medical History: Obstetric history affecting care in pregnancy	No<br>Yes
pmh_PRG023	Past Medical History: Complications specified during childbirth	No<br>Yes
pmh_PRG027	Past Medical History: Complications specified during the puerperium	No<br>Yes

© 2025 Wong AH et al. *JAMA Network Open*.

pmh_PRG028	Past Medical History: Other specified complications in pregnancy	No<br>Yes
pmh_PRG019	Past Medical History: Diabetes or abnormal glucose tolerance complicating pregnancy; childbirth; or the puerperium	No<br>Yes
pmh_PRG016	Past Medical History: Previous C-section	No<br>Yes
pmh_GEN022	Past Medical History: Benign ovarian cyst	No<br>Yes
pmh_PRG008	Past Medical History: Supervision of high-risk pregnancy	No<br>Yes
pmh_INJ037	Past Medical History: Complication of other surgical or medical care, injury, initial encounter	No<br>Yes
pmh_DIG001	Past Medical History: Intestinal infection	No<br>Yes
pmh_SKN005	Past Medical History: Contact dermatitis	No<br>Yes
pmh_SKN002	Past Medical History: Other specified inflammatory condition of skin	No<br>Yes
pmh_INF007	Past Medical History: Hepatitis	No<br>Yes
pmh_SKN007	Past Medical History: Other specified and unspecified skin disorders	No<br>Yes
pmh_SKN003	Past Medical History: Pressure ulcer of skin	No<br>Yes
pmh_SKN004	Past Medical History: Non-pressure ulcer of skin	No<br>Yes
pmh_MUS001	Past Medical History: Infective arthritis	No<br>Yes
pmh_MUS003	Past Medical History: Rheumatoid arthritis and related disease	No<br>Yes
pmh_MUS007	Past Medical History: Other specified joint disorders	No<br>Yes
pmh_MUS010	Past Medical History: Musculoskeletal pain, not low back pain	No<br>Yes
pmh_MUS011	Past Medical History: Spondylopathies/spondyloarthropathy (including infective)	No<br>Yes
pmh_MUS038	Past Medical History: Low back pain	No<br>Yes

© 2025 Wong AH et al. *JAMA Network Open*.

pmh_MUS025	Past Medical History: Other specified connective tissue disease	No<br>Yes
pmh_MUS009	Past Medical History: Tendon and synovial disorders	No<br>Yes
pmh_MUS026	Past Medical History: Muscle disorders	No<br>Yes
pmh_SYM016	Past Medical History: Other general signs and symptoms	No<br>Yes
pmh_MUS002	Past Medical History: Osteomyelitis	No<br>Yes
pmh_MUS030	Past Medical History: Aseptic necrosis and osteonecrosis	No<br>Yes
pmh_MUS016	Past Medical History: Stress fracture, initial encounter	No<br>Yes
pmh_MUS021	Past Medical History: Acquired foot deformities	No<br>Yes
pmh_MUS023	Past Medical History: Acquired deformities (excluding foot)	No<br>Yes
pmh_MUS012	Past Medical History: Biomechanical lesions	No<br>Yes
pmh_MAL001	Past Medical History: Cardiac and circulatory congenital anomalies	No<br>Yes
pmh_MAL006	Past Medical History: Cleft lip or palate	No<br>Yes
pmh_MAL002	Past Medical History: Digestive congenital anomalies	No<br>Yes
pmh_MAL003	Past Medical History: Genitourinary congenital anomalies	No<br>Yes
pmh_MAL008	Past Medical History: Musculoskeletal congenital conditions	No<br>Yes
pmh_MAL009	Past Medical History: Chromosomal abnormalities	No<br>Yes
pmh_PNL010	Past Medical History: Newborn affected by maternal conditions or complications of labor/delivery	No<br>Yes
pmh_PNL002	Past Medical History: Short gestation; low birth weight; and fetal growth retardation	No<br>Yes
pmh_PRG030	Past Medical History: Maternal outcome of delivery	No<br>Yes

© 2025 Wong AH et al. *JAMA Network Open*.

pmh_PNL003	Past Medical History: Neonatal acidemia and hypoxia	No<br>Yes
pmh_PNL006	Past Medical History: Respiratory perinatal condition	No<br>Yes
pmh_SYM001	Past Medical History: Syncope	No<br>Yes
pmh_SYM010	Past Medical History: Nervous system signs and symptoms	No<br>Yes
pmh_SYM014	Past Medical History: Skin/Subcutaneous signs and symptoms	No<br>Yes
pmh_SYM012	Past Medical History: Circulatory signs and symptoms	No<br>Yes
pmh_SYM013	Past Medical History: Respiratory signs and symptoms	No<br>Yes
pmh_SYM005	Past Medical History: Dysphagia	No<br>Yes
pmh_GEN008	Past Medical History: Urinary incontinence	No<br>Yes
pmh_SYM006	Past Medical History: Abdominal pain and other digestive/abdomen signs and symptoms	No<br>Yes
pmh_SYM011	Past Medical History: Genitourinary signs and symptoms	No<br>Yes
pmh_NEO074	Past Medical History: Conditions due to neoplasm or the treatment of neoplasm	No<br>Yes
pmh_SYM017	Past Medical History: Abnormal findings without diagnosis	No<br>Yes
pmh_INJ001	Past Medical History: Fracture of head and neck, initial encounter	No<br>Yes
pmh_INJ002	Past Medical History: Fracture of the spine and back, initial encounter	No<br>Yes
pmh_INJ004	Past Medical History: Fracture of the upper limb, initial encounter	No<br>Yes
pmh_INJ006	Past Medical History: Fracture of the neck of the femur (hip), initial encounter	No<br>Yes
pmh_INJ024	Past Medical History: Sprains and strains, initial encounter	No<br>Yes
pmh_INJ027	Past Medical History: Other unspecified injury	No<br>Yes

© 2025 Wong AH et al. *JAMA Network Open*.

pmh_INJ010	Past Medical History: Internal organ injury, initial encounter	No<br>Yes
pmh_INJ013	Past Medical History: Open wounds of trunk, initial encounter	No<br>Yes
pmh_FAC021	Past Medical History: Personal/family history of disease	No<br>Yes
pmh_MBD017	Past Medical History: Alcohol-related disorders	No<br>Yes
pmh_PNL009	Past Medical History: Perinatal infections	No<br>Yes
pmh_INJ073	Past Medical History: Injury, sequela	No<br>Yes
pmh_INF010	Past Medical History: Sexually transmitted infections (excluding HIV and hepatitis)	No<br>Yes
pmh_INJ019	Past Medical History: Burn and corrosion, initial encounter	No<br>Yes
pmh_INJ026	Past Medical History: Other specified injury	No<br>Yes
pmh_MBD012	Past Medical History: Suicidal ideation/attempt/intentional self-harm	No<br>Yes
pmh_INJ023	Past Medical History: Toxic effects, initial encounter	No<br>Yes
pmh_INJ021	Past Medical History: Effect of other external causes, initial encounter	No<br>Yes
pmh_MUS008	Past Medical History: Immune-mediated/reactive arthropathies	No<br>Yes
pmh_INJ028	Past Medical History: Adverse effects of drugs and medicaments, initial encounter	No<br>Yes
pmh_INJ031	Past Medical History: Allergic reactions	No<br>Yes
pmh_INJ032	Past Medical History: Maltreatment/abuse	No<br>Yes
pmh_INJ036	Past Medical History: Complication of transplanted organs or tissue, initial encounter	No<br>Yes
pmh_CIR038	Past Medical History: Postprocedural or postoperative circulatory system complication	No<br>Yes
pmh_FAC009	Past Medical History: Implant, device or graft related encounter	No<br>Yes

© 2025 Wong AH et al. *JAMA Network Open*.

pmh_NVS001	Past Medical History: Meningitis	No<br>Yes
pmh_NVS002	Past Medical History: Encephalitis	No<br>Yes
pmh_EYE012	Past Medical History: Other specified eye disorders	No<br>Yes
pmh_NVS003	Past Medical History: Other specified CNS infection and poliomyelitis	No<br>Yes
pmh_GEN013	Past Medical History: Inflammatory conditions of male genital organs	No<br>Yes
pmh_GEN004	Past Medical History: Urinary tract infections	No<br>Yes
pmh_NVS014	Past Medical History: CNS abscess	No<br>Yes
pmh_INF011	Past Medical History: Sequela of specified infectious disease conditions	No<br>Yes
pmh_NEO002	Past Medical History: Head and neck cancers - lip and oral cavity	No<br>Yes
pmh_NEO004	Past Medical History: Head and neck cancers - salivary gland	No<br>Yes
pmh_NEO009	Past Medical History: Head and neck cancers - tonsils	No<br>Yes
pmh_NEO003	Past Medical History: Head and neck cancers - throat	No<br>Yes
pmh_NEO005	Past Medical History: Head and neck cancers - nasopharyngeal	No<br>Yes
pmh_NEO006	Past Medical History: Head and neck cancers - hypopharyngeal	No<br>Yes
pmh_NEO007	Past Medical History: Head and neck cancers - pharyngeal	No<br>Yes
pmh_NEO010	Past Medical History: Head and neck cancers - all other types	No<br>Yes
pmh_NEO012	Past Medical History: Gastrointestinal cancers - esophagus	No<br>Yes
pmh_NEO013	Past Medical History: Gastrointestinal cancers - stomach	No<br>Yes
pmh_NEO014	Past Medical History: Gastrointestinal cancers - small intestine	No<br>Yes

© 2025 Wong AH et al. *JAMA Network Open*.

pmh_NEO016	Past Medical History: Gastrointestinal cancers - anus	No<br>Yes
pmh_NEO017	Past Medical History: Gastrointestinal cancers - liver	No<br>Yes
pmh_NEO018	Past Medical History: Gastrointestinal cancers - bile duct	No<br>Yes
pmh_NEO019	Past Medical History: Gastrointestinal cancers - gallbladder	No<br>Yes
pmh_NEO021	Past Medical History: Gastrointestinal cancers - all other types	No<br>Yes
pmh_NEO008	Past Medical History: Head and neck cancers - laryngeal	No<br>Yes
pmh_NEO052	Past Medical History: Endocrine system cancers - thymus	No<br>Yes
pmh_NEO011	Past Medical History: Cardiac cancers	No<br>Yes
pmh_NEO023	Past Medical History: Bone cancer	No<br>Yes
pmh_NEO027	Past Medical History: Skin cancers - squamous cell carcinoma	No<br>Yes
pmh_NEO067	Past Medical History: Mesothelioma	No<br>Yes
pmh_NEO020	Past Medical History: Gastrointestinal cancers - peritoneum	No<br>Yes
pmh_NEO038	Past Medical History: Female reproductive system cancers - all other types	No<br>Yes
pmh_NEO036	Past Medical History: Female reproductive system cancers - vulva	No<br>Yes
pmh_NEO037	Past Medical History: Female reproductive system cancers - vagina	No<br>Yes
pmh_NEO032	Past Medical History: Female reproductive system cancers - cervix	No<br>Yes
pmh_NEO035	Past Medical History: Female reproductive system cancers - endometrium	No<br>Yes
pmh_NEO033	Past Medical History: Female reproductive system cancers - ovary	No<br>Yes
pmh_NEO034	Past Medical History: Female reproductive system cancers - fallopian tube	No<br>Yes

© 2025 Wong AH et al. *JAMA Network Open*.

pmh_NEO041	Past Medical History: Male reproductive system cancers - penis	No<br>Yes
pmh_NEO039	Past Medical History: Male reproductive system cancers - prostate	No<br>Yes
pmh_NEO040	Past Medical History: Male reproductive system cancers - testis	No<br>Yes
pmh_NEO045	Past Medical History: Urinary system cancers - kidney	No<br>Yes
pmh_NEO044	Past Medical History: Urinary system cancers - ureter and renal pelvis	No<br>Yes
pmh_NEO046	Past Medical History: Urinary system cancers - urethra	No<br>Yes
pmh_NEO001	Past Medical History: Head and neck cancers - eye	No<br>Yes
pmh_NEO050	Past Medical History: Endocrine system cancers - thyroid	No<br>Yes
pmh_NEO053	Past Medical History: Endocrine system cancers - adrenocortical	No<br>Yes
pmh_NEO056	Past Medical History: Endocrine system cancers - all other types	No<br>Yes
pmh_NEO054	Past Medical History: Endocrine system cancers - parathyroid	No<br>Yes
pmh_NEO055	Past Medical History: Endocrine system cancers - pituitary gland	No<br>Yes
pmh_NEO069	Past Medical History: Cancer of other sites	No<br>Yes
pmh_NEO057	Past Medical History: Hodgkin lymphoma	No<br>Yes
pmh_NEO065	Past Medical History: Multiple myeloma	No<br>Yes
pmh_NEO059	Past Medical History: Leukemia - acute lymphoblastic leukemia (ALL)	No<br>Yes
pmh_NEO063	Past Medical History: Leukemia - hairy cell	No<br>Yes
pmh_NEO060	Past Medical History: Leukemia - acute myeloid leukemia (AML)	No<br>Yes
pmh_NEO062	Past Medical History: Leukemia - chronic myeloid leukemia (CML)	No<br>Yes

© 2025 Wong AH et al. *JAMA Network Open*.

pmh_NEO029	Past Medical History: Breast cancer - ductal carcinoma in situ (DCIS)	No<br>Yes
pmh_NEO068	Past Medical History: Myelodysplastic syndrome (MDS)	No<br>Yes
pmh_BLD001	Past Medical History: Nutritional anemia	No<br>Yes
pmh_BLD007	Past Medical History: Diseases of white blood cells	No<br>Yes
pmh_BLD008	Past Medical History: Immunity disorders	No<br>Yes
pmh_END008	Past Medical History: Malnutrition	No<br>Yes
pmh_END007	Past Medical History: Nutritional deficiencies	No<br>Yes
pmh_END017	Past Medical History: Sequela of malnutrition and other nutritional deficiencies	No<br>Yes
pmh_END009	Past Medical History: Obesity	No<br>Yes
pmh_EXT007	Past Medical History: External cause codes: motor vehicle traffic (MVT); initial encounter	No<br>Yes
pmh_END012	Past Medical History: Cystic fibrosis	No<br>Yes
pmh_FAC025	Past Medical History: Other specified status	No<br>Yes
pmh_END014	Past Medical History: Postprocedural or postoperative endocrine or metabolic complication	No<br>Yes
pmh_EXT001	Past Medical History: External cause codes: cut/pierce; initial encounter	No<br>Yes
pmh_MBD004	Past Medical History: Other specified and unspecified mood disorders	No<br>Yes
pmh_MBD002	Past Medical History: Depressive disorders	No<br>Yes
pmh_MBD003	Past Medical History: Bipolar and related disorders	No<br>Yes
pmh_MBD026	Past Medical History: Mental and substance use disorders in remission	No<br>Yes
pmh_MBD018	Past Medical History: Opioid-related disorders	No<br>Yes

© 2025 Wong AH et al. *JAMA Network Open*.

pmh_MBD022	Past Medical History: Hallucinogen-related disorders	No<br>Yes
pmh_MBD024	Past Medical History: Tobacco-related disorders	No<br>Yes
pmh_MBD006	Past Medical History: Obsessive-compulsive and related disorders	No<br>Yes
pmh_MBD011	Past Medical History: Somatic disorders	No<br>Yes
pmh_MBD008	Past Medical History: Disruptive, impulse-control and conduct disorders	No<br>Yes
pmh_NVS004	Past Medical History: Parkinson`s disease	No<br>Yes
pmh_NVS005	Past Medical History: Multiple sclerosis	No<br>Yes
pmh_CIR020	Past Medical History: Cerebral infarction	No<br>Yes
pmh_NVS012	Past Medical History: Transient cerebral ischemia	No<br>Yes
pmh_NVS022	Past Medical History: Sequela of specified nervous system conditions	No<br>Yes
pmh_NVS019	Past Medical History: Nervous system pain and pain syndromes	No<br>Yes
pmh_NVS013	Past Medical History: Coma; stupor; and brain damage	No<br>Yes
pmh_NVS021	Past Medical History: Postprocedural or postoperative nervous system complication	No<br>Yes
pmh_EYE004	Past Medical History: Uveitis and ocular inflammation	No<br>Yes
pmh_EYE007	Past Medical History: Strabismus	No<br>Yes
pmh_EYE009	Past Medical History: Refractive error	No<br>Yes
pmh_EYE011	Past Medical History: Postprocedural or postoperative eye complication	No<br>Yes
pmh_EAR001	Past Medical History: Otitis media	No<br>Yes
pmh_EAR003	Past Medical History: Diseases of inner ear and related conditions	No<br>Yes

© 2025 Wong AH et al. *JAMA Network Open*.

pmh_CIR008	Past Medical History: Hypertension with complications and secondary hypertension	No<br>Yes
pmh_GEN003	Past Medical History: Chronic kidney disease	No<br>Yes
pmh_CIR009	Past Medical History: Acute myocardial infarction	No<br>Yes
pmh_CIR010	Past Medical History: Complications of acute myocardial infarction	No<br>Yes
pmh_CIR015	Past Medical History: Other and ill-defined heart disease	No<br>Yes
pmh_CIR027	Past Medical History: Arterial dissections	No<br>Yes
pmh_CIR013	Past Medical History: Acute pulmonary embolism	No<br>Yes
pmh_CIR014	Past Medical History: Pulmonary heart disease	No<br>Yes
pmh_CIR016	Past Medical History: Conduction disorders	No<br>Yes
pmh_CIR022	Past Medical History: Sequela of hemorrhagic cerebrovascular disease	No<br>Yes
pmh_CIR030	Past Medical History: Aortic and peripheral arterial embolism or thrombosis	No<br>Yes
pmh_CIR035	Past Medical History: Varicose veins of lower extremity	No<br>Yes
pmh_CIR036	Past Medical History: Post thrombotic syndrome and venous insufficiency/hypertension	No<br>Yes
pmh_CIR028	Past Medical History: Gangrene	No<br>Yes
pmh_RSP001	Past Medical History: Sinusitis	No<br>Yes
pmh_RSP004	Past Medical History: Acute and chronic tonsillitis	No<br>Yes
pmh_INF012	Past Medical History: COVID-19	No<br>Yes
pmh_RSP005	Past Medical History: Acute bronchitis	No<br>Yes
pmh_RSP015	Past Medical History: Mediastinal disorders	No<br>Yes

© 2025 Wong AH et al. *JAMA Network Open*.

pmh_RSP011	Past Medical History: Pleurisy, pleural effusion and pulmonary collapse	No<br>Yes
pmh_RSP014	Past Medical History: Pneumothorax	No<br>Yes
pmh_RSP017	Past Medical History: Postprocedural or postoperative respiratory system complication	No<br>Yes
pmh_DIG002	Past Medical History: Disorders of teeth and gingiva	No<br>Yes
pmh_DIG006	Past Medical History: Gastrointestinal and biliary perforation	No<br>Yes
pmh_DIG021	Past Medical History: Gastrointestinal hemorrhage	No<br>Yes
pmh_DIG022	Past Medical History: Noninfectious gastroenteritis	No<br>Yes
pmh_DIG023	Past Medical History: Noninfectious hepatitis	No<br>Yes
pmh_DIG024	Past Medical History: Postprocedural or postoperative digestive system complication	No<br>Yes
pmh_SKN006	Past Medical History: Postprocedural or postoperative skin complication	No<br>Yes
pmh_MUS036	Past Medical History: Autoinflammatory syndromes	No<br>Yes
pmh_MUS004	Past Medical History: Juvenile arthritis	No<br>Yes
pmh_MUS034	Past Medical History: Crystal arthropathies (excluding gout)	No<br>Yes
pmh_MUS005	Past Medical History: Other specified chronic arthropathy	No<br>Yes
pmh_MUS031	Past Medical History: Traumatic arthropathy	No<br>Yes
pmh_MUS032	Past Medical History: Neurogenic/neuropathic arthropathy	No<br>Yes
pmh_MUS024	Past Medical History: Systemic lupus erythematosus and connective tissue disorders	No<br>Yes
pmh_MUS022	Past Medical History: Scoliosis and other postural dorsopathic deformities	No<br>Yes
pmh_MUS017	Past Medical History: Stress fracture, subsequent encounter	No<br>Yes

© 2025 Wong AH et al. *JAMA Network Open*.

pmh_MUS020	Past Medical History: Pathological, stress and atypical fractures, sequela	No<br>Yes
pmh_MUS027	Past Medical History: Musculoskeletal abscess	No<br>Yes
pmh_MUS013	Past Medical History: Osteoporosis	No<br>Yes
pmh_MUS015	Past Medical History: Pathological fracture, subsequent encounter	No<br>Yes
pmh_MUS035	Past Medical History: Osteomalacia	No<br>Yes
pmh_MUS014	Past Medical History: Pathological fracture, initial encounter	No<br>Yes
pmh_MUS019	Past Medical History: Atypical fracture, subsequent encounter	No<br>Yes
pmh_MUS028	Past Medical History: Other specified bone disease and musculoskeletal deformities	No<br>Yes
pmh_MUS037	Past Medical History: Postprocedural or postoperative musculoskeletal system complication	No<br>Yes
pmh_INJ005	Past Medical History: Fracture of the lower limb (except hip), initial encounter	No<br>Yes
pmh_INJ042	Past Medical History: Fracture of lower limb (except hip), subsequent encounter	No<br>Yes
pmh_GEN009	Past Medical History: Hematuria	No<br>Yes
pmh_GEN010	Past Medical History: Proteinuria	No<br>Yes
pmh_GEN011	Past Medical History: Vesicoureteral reflux	No<br>Yes
pmh_GEN012	Past Medical History: Hyperplasia of prostate	No<br>Yes
pmh_GEN016	Past Medical History: Other specified male genital disorders	No<br>Yes
pmh_GEN015	Past Medical History: Male infertility	No<br>Yes
pmh_GEN014	Past Medical History: Erectile dysfunction	No<br>Yes
pmh_GEN020	Past Medical History: Prolapse of female genital organs	No<br>Yes

© 2025 Wong AH et al. *JAMA Network Open*.

pmh_GEN021	Past Medical History: Menstrual disorders	No<br>Yes
pmh_GEN026	Past Medical History: Postprocedural or postoperative genitourinary system complication	No<br>Yes
pmh_PRG005	Past Medical History: Ectopic pregnancy and complications of ectopic pregnancy	No<br>Yes
pmh_PRG003	Past Medical History: Spontaneous abortion and complications of spontaneous abortion	No<br>Yes
pmh_PRG007	Past Medical History: Complications following ectopic and/or molar pregnancy	No<br>Yes
pmh_PRG010	Past Medical History: Hemorrhage after first trimester	No<br>Yes
pmh_PRG011	Past Medical History: Early or threatened labor	No<br>Yes
pmh_PRG025	Past Medical History: Anesthesia complications during pregnancy	No<br>Yes
pmh_PRG012	Past Medical History: Multiple gestation	No<br>Yes
pmh_PRG024	Past Medical History: Malposition, disproportion or other labor complications	No<br>Yes
pmh_PRG017	Past Medical History: Maternal care for abnormality of pelvic organs	No<br>Yes
pmh_PRG013	Past Medical History: Maternal care related to fetal conditions	No<br>Yes
pmh_PRG014	Past Medical History: Polyhydramnios and other problems of amniotic cavity	No<br>Yes
pmh_PRG021	Past Medical History: Maternal intrauterine infection	No<br>Yes
pmh_PRG029	Past Medical History: Uncomplicated pregnancy, delivery or puerperium	No<br>Yes
pmh_PRG018	Past Medical History: Maternal care related to disorders of the placenta and placental implantation	No<br>Yes
pmh_PRG022	Past Medical History: Prolonged pregnancy	No<br>Yes
pmh_PRG026	Past Medical History: OB-related trauma to perineum and vulva	No<br>Yes
pmh_FAC024	Past Medical History: Carrier status	No<br>Yes

© 2025 Wong AH et al. *JAMA Network Open*.

pmh_PNL014	Past Medical History: Neonatal abstinence syndrome	No<br>Yes
pmh_PNL015	Past Medical History: Fetal alcohol syndrome	No<br>Yes
pmh_PNL013	Past Medical History: Other specified and unspecified perinatal conditions	No<br>Yes
pmh_PNL008	Past Medical History: Birth trauma	No<br>Yes
pmh_PNL005	Past Medical History: Respiratory distress syndrome	No<br>Yes
pmh_PNL011	Past Medical History: Hemorrhagic and hematologic disorders of newborn	No<br>Yes
pmh_PNL007	Past Medical History: Hemolytic jaundice and perinatal jaundice	No<br>Yes
pmh_PNL012	Past Medical History: Neonatal digestive and feeding disorders	No<br>Yes
pmh_PNL004	Past Medical History: Neonatal cerebral disorders	No<br>Yes
pmh_MAL004	Past Medical History: Nervous system congenital anomalies	No<br>Yes
pmh_MAL005	Past Medical History: Congenital malformations of eye, ear, face, neck	No<br>Yes
pmh_MAL007	Past Medical History: Respiratory congenital malformations	No<br>Yes
pmh_CIR012	Past Medical History: Nonspecific chest pain	No<br>Yes
pmh_SYM004	Past Medical History: Nausea and vomiting	No<br>Yes
pmh_SYM015	Past Medical History: General sensation/perception signs and symptoms	No<br>Yes
pmh_SYM002	Past Medical History: Fever	No<br>Yes
pmh_SYM007	Past Medical History: Malaise and fatigue	No<br>Yes
pmh_SYM003	Past Medical History: Shock	No<br>Yes
pmh_SYM009	Past Medical History: Abnormal findings related to substance use	No<br>Yes

© 2025 Wong AH et al. *JAMA Network Open*.

pmh_INJ017	Past Medical History: Superficial injury; contusion, initial encounter	No<br>Yes
pmh_INJ054	Past Medical History: Superficial injury; contusion, subsequent encounter	No<br>Yes
pmh_INJ011	Past Medical History: Open wounds of head and neck, initial encounter	No<br>Yes
pmh_INJ048	Past Medical History: Open wounds of head and neck, subsequent encounter	No<br>Yes
pmh_INJ038	Past Medical History: Fracture of head and neck, subsequent encounter	No<br>Yes
pmh_INJ007	Past Medical History: Dislocations, initial encounter	No<br>Yes
pmh_INJ044	Past Medical History: Dislocations, subsequent encounter	No<br>Yes
pmh_INJ025	Past Medical History: Injury to nerves, muscles and tendons, initial encounter	No<br>Yes
pmh_INJ062	Past Medical History: Injury to nerves, muscles and tendons, subsequent encounter	No<br>Yes
pmh_INJ063	Past Medical History: Other specified injury, subsequent encounter	No<br>Yes
pmh_INJ064	Past Medical History: Other unspecified injuries, subsequent encounter	No<br>Yes
pmh_INJ008	Past Medical History: Traumatic brain injury (TBI); concussion, initial encounter	No<br>Yes
pmh_INJ045	Past Medical History: Traumatic brain injury (TBI); concussion, subsequent encounter	No<br>Yes
pmh_INJ018	Past Medical History: Crushing injury, initial encounter	No<br>Yes
pmh_INJ039	Past Medical History: Fracture of the spine and back, subsequent encounter	No<br>Yes
pmh_INJ061	Past Medical History: Sprains and strains, subsequent encounter	No<br>Yes
pmh_INJ009	Past Medical History: Spinal cord injury (SCI), initial encounter	No<br>Yes
pmh_INJ046	Past Medical History: Spinal cord injury (SCI), subsequent encounter	No<br>Yes
pmh_INJ016	Past Medical History: Injury to blood vessels, initial encounter	No<br>Yes

© 2025 Wong AH et al. *JAMA Network Open*.

pmh_INJ053	Past Medical History: Injury to blood vessels, subsequent encounter	No<br>Yes
pmh_INJ050	Past Medical History: Open wounds of trunk, subsequent encounter	No<br>Yes
pmh_INJ003	Past Medical History: Fracture of torso, initial encounter	No<br>Yes
pmh_INJ040	Past Medical History: Fracture of torso, subsequent encounter	No<br>Yes
pmh_INJ047	Past Medical History: Internal organ injury, subsequent encounter	No<br>Yes
pmh_INJ012	Past Medical History: Open wounds to limbs, initial encounter	No<br>Yes
pmh_INJ049	Past Medical History: Open wounds to limbs, subsequent encounter	No<br>Yes
pmh_INJ041	Past Medical History: Fracture of the upper limb, subsequent encounter	No<br>Yes
pmh_INJ055	Past Medical History: Crushing injury, subsequent encounter	No<br>Yes
pmh_INJ014	Past Medical History: Amputation of a limb, initial encounter	No<br>Yes
pmh_INJ051	Past Medical History: Amputation of a limb, subsequent encounter	No<br>Yes
pmh_INJ043	Past Medical History: Fracture of the neck of the femur (hip), subsequent encounter	No<br>Yes
pmh_INJ020	Past Medical History: Effect of foreign body entering opening, initial encounter	No<br>Yes
pmh_INJ057	Past Medical History: Effect of foreign body entering opening, subsequent encounter	No<br>Yes
pmh_INJ056	Past Medical History: Burns and corrosion, subsequent encounter	No<br>Yes
pmh_INJ058	Past Medical History: Effect of other external causes, subsequent encounter	No<br>Yes
pmh_INJ022	Past Medical History: Poisoning by drugs, initial encounter	No<br>Yes
pmh_INJ065	Past Medical History: Adverse effects of drugs and medicaments, subsequent encounter	No<br>Yes
pmh_INJ075	Past Medical History: Poisoning/toxic effect/adverse effects/underdosing, sequela	No<br>Yes

© 2025 Wong AH et al. *JAMA Network Open*.

pmh_MBD027	Past Medical History: Suicide attempt/intentional self-harm; subsequent encounter	No<br>Yes
pmh_MBD034	Past Medical History: Mental and substance use disorders; sequela	No<br>Yes
pmh_INJ059	Past Medical History: Poisoning by drugs, subsequent encounter	No<br>Yes
pmh_INJ060	Past Medical History: Toxic effects, subsequent encounter	No<br>Yes
pmh_INJ074	Past Medical History: Effect of other external causes, sequela	No<br>Yes
pmh_INJ068	Past Medical History: Maltreatment/abuse, subsequent encounter	No<br>Yes
pmh_INJ067	Past Medical History: Allergic reactions, subsequent encounter	No<br>Yes
pmh_INJ076	Past Medical History: Complication, sequela	No<br>Yes
pmh_INJ072	Past Medical History: Complication of other surgical or medical care, injury, subsequent encounter	No<br>Yes
pmh_INJ033	Past Medical History: Complication of cardiovascular device, implant or graft, initial encounter	No<br>Yes
pmh_INJ069	Past Medical History: Complication of cardiovascular device, implant or graft, subsequent encounter	No<br>Yes
pmh_INJ034	Past Medical History: Complication of genitourinary device, implant or graft, initial encounter	No<br>Yes
pmh_INJ070	Past Medical History: Complication of genitourinary device, implant or graft, subsequent encounter	No<br>Yes
pmh_INJ035	Past Medical History: Complication of internal orthopedic device or implant, initial encounter	No<br>Yes
pmh_INJ071	Past Medical History: Complication of internal orthopedic device or implant, subsequent encounter	No<br>Yes
pmh_EXT016	Past Medical History: External cause codes: struck by; against; initial encounter	No<br>Yes
pmh_EXT003	Past Medical History: External cause codes: fall; initial encounter	No<br>Yes
pmh_EXT018	Past Medical History: External cause codes: other specified, classifiable and NEC; initial encounter	No<br>Yes
pmh_EXT029	Past Medical History: External cause codes: subsequent encounter	No<br>Yes

© 2025 Wong AH et al. *JAMA Network Open*.

pmh_EXT030	Past Medical History: External cause codes: sequela	No<br>Yes
pmh_EXT009	Past Medical History: External cause codes: pedestrian; not MVT; initial encounter	No<br>Yes
pmh_FAC016	Past Medical History: Exposure, encounters, screening or contact with infectious disease	No<br>Yes
pmh_EXT008	Past Medical History: External cause codes: pedal cyclist; not MVT; initial encounter	No<br>Yes
pmh_FAC012	Past Medical History: Other specified encounters and counseling	No<br>Yes
pmh_EXT010	Past Medical History: External cause codes: transport; not MVT; initial encounter	No<br>Yes
pmh_FAC013	Past Medical History: Contraceptive and procreative management	No<br>Yes
pmh_PNL001	Past Medical History: Liveborn	No<br>Yes
pmh_FAC010	Past Medical History: Other aftercare encounter	No<br>Yes
pmh_FAC006	Past Medical History: Encounter for antineoplastic therapies	No<br>Yes
pmh_FAC019	Past Medical History: Socioeconomic/psychosocial factors	No<br>Yes
pmh_FAC001	Past Medical History: Encounter for administrative purposes	No<br>Yes
pmh_FAC014	Past Medical History: Medical examination/evaluation	No<br>Yes
pmh_FAC003	Past Medical History: Encounter for observation and examination for conditions ruled out (excludes infectious disease, neoplasm, mental disorders)	No<br>Yes
pmh_FAC008	Past Medical History: Neoplasm-related encounters	No<br>Yes
pmh_EXT002	Past Medical History: External cause codes: drowning/submersion; initial encounter	No<br>Yes
pmh_EXT006	Past Medical History: External cause codes: machinery; initial encounter	No<br>Yes
pmh_EXT005	Past Medical History: External cause codes: firearm; initial encounter	No<br>Yes
pmh_EXT012	Past Medical History: External cause codes: bites; initial encounter	No<br>Yes

© 2025 Wong AH et al. *JAMA Network Open*.

pmh_EXT011	Past Medical History: External cause codes: natural/environment; initial encounter	No<br>Yes
pmh_EXT004	Past Medical History: External cause codes: fire/burn; initial encounter	No<br>Yes
pmh_EXT013	Past Medical History: External cause codes: overexertion; initial encounter	No<br>Yes
pmh_EXT019	Past Medical History: External cause codes: unspecified mechanism	No<br>Yes
pmh_EXT028	Past Medical History: External cause codes: evidence of alcohol involvement	No<br>Yes
pmh_EXT027	Past Medical History: External cause codes: place of occurrence of the external cause	No<br>Yes
pmh_EXT026	Past Medical History: External cause codes: activity codes	No<br>Yes
pmh_DEN001	Past Medical History: Any dental condition including traumatic injury	No<br>Yes
pmh_FAC007	Past Medical History: Encounter for mental health conditions	No<br>Yes
pmh_FAC018	Past Medical History: Screening for neurocognitive or neurodevelopmental condition	No<br>Yes
pmh_FAC015	Past Medical History: Resistance to antimicrobial drugs	No<br>Yes
pmh_FAC017	Past Medical History: No immunization or under immunization	No<br>Yes
pmh_FAC005	Past Medical History: Encounter for prophylactic measures (excludes immunization)	No<br>Yes
pmh_PRG001	Past Medical History: Antenatal screening	No<br>Yes
pmh_PRG002	Past Medical History: Gestational weeks	No<br>Yes
pmh_FAC004	Past Medical History: Encounter for prophylactic or other procedures	No<br>Yes
pmh_FAC002	Past Medical History: Encounter for mental health services related to abuse	No<br>Yes
pmh_FAC011	Past Medical History: Counseling related to sexual behavior or orientation	No<br>Yes
pmh_FAC020	Past Medical History: Lifestyle/life management factors	No<br>Yes
**Outpatient medications (ATC classifications - https://www.who.int/tools/atc-ddd-toolkit/atc-classification)**		

© 2025 Wong AH et al. *JAMA Network Open*.

opt_med_THYROID PREPS	OPT Medication: Thyroid Preps	0<br>1
opt_med_ANALGESICS	OPT Medication: Analgesics	0<br>1
opt_med_ANTIHYPERGLYCEMICS	OPT Medication: Antihyperglycemics	0<br>1
opt_med_ANTIBIOTICS	OPT Medication: Antibiotics	0<br>1
opt_med_CNS DRUGS	OPT Medication: CNS Drugs	0<br>1
opt_med_HORMONES	OPT Medication: Hormones	0<br>1
opt_med_UNCLASSIFIED DRUG PRODUCTS	OPT Medication: Unclassified Drug Products	0<br>1
opt_med_PSYCHOTHERAPEUTIC DRUGS	OPT Medication: Psychotherapeutic Drugs	0<br>1
opt_med_VITAMINS	OPT Medication: Vitamins	0<br>1
opt_med_CARDIOVASCULAR	OPT Medication: Cardiovascular	0<br>1
opt_med_CARDIAC DRUGS	OPT Medication: Cardiac Drugs	0<br>1
opt_med_EENT PREPS	OPT Medication: EENT Preps	0<br>1
opt_med_ANTICOAGULANTS	OPT Medication: Anticoagulants	0<br>1
opt_med_ELECT/CALORIC/H2O	OPT Medication: Elect/Caloric/H2O	0<br>1
opt_med_GASTROINTESTINAL	OPT Medication: Gastrointestinal	0<br>1
opt_med_ANTINEOPLASTICS	OPT Medication: Antineoplastics	0<br>1
opt_med_ANTIPLATELET DRUGS	OPT Medication: Antiplatelet Drugs	0<br>1
opt_med_ANTIARTHRITICS	OPT Medication: Antiarthritics	0<br>1
opt_med_ANTIFUNGALS	OPT Medication: Antifungals	0<br>1
opt_med_DIURETICS	OPT Medication: Diuretics	0

© 2025 Wong AH et al. *JAMA Network Open*.

		1
opt_med_*Unspecified	OPT Medication: Unspecified	0<br>1
opt_med_MISCELLANEOUS MEDICAL SUPPLIES, DEVICES, NON-DRUG	OPT Medication: Miscellaneous Medical Supplies, Devices, Non-Drug	0<br>1
opt_med_ANESTHETICS	OPT Medication: Anesthetics	0<br>1
opt_med_SKIN PREPS	OPT Medication: Skin Preps	0<br>1
opt_med_ANTIHISTAMINES	OPT Medication: Antihistamines	0<br>1
opt_med_ANTIASTHMATICS	OPT Medication: Antiasthmatics	0<br>1
opt_med_MUSCLE RELAXANTS	OPT Medication: Muscle Relaxants	0<br>1
opt_med_SEDATIVE/HYPNOTICS	OPT Medication: Sedative/Hypnotics	0<br>1
opt_med_SMOKING DETERRENTS	OPT Medication: Smoking Deterrents	0<br>1
opt_med_COUGH/COLD PREPARATIONS	OPT Medication: Cough/Cold Preparations	0<br>1
opt_med_DIAGNOSTIC	OPT Medication: Diagnostic	0<br>1
opt_med_BIOLOGICALS	OPT Medication: Biologicals	0<br>1
opt_med_ANTIVIRALS	OPT Medication: Antivirals	0<br>1
opt_med_AUTONOMIC DRUGS	OPT Medication: Autonomic Drugs	0<br>1
opt_med_ANTIPARASITICS	OPT Medication: Antiparasitic	0<br>1
opt_med_CONTRACEPTIVES	OPT Medication: Contraceptives	0<br>1
opt_med_ANTIDOTES	OPT Medication: Antidotes	0<br>1
opt_med_PRE-NATAL VITAMINS	OPT Medication: Pre-Natal Vitamins	0<br>1
opt_med_HERBALS	OPT Medication: Herbals	0<br>1

© 2025 Wong AH et al. *JAMA Network Open*.

opt_med_ANTIPARKINSON DRUGS	OPT Medication: Anti-Parkinson Drugs	0<br>1
opt_med_IMMUNOSUPPRESSANTS	OPT Medication: Immunosuppressants	0<br>1
opt_med_ANTIINFECTIVES/MISCELLANEOUS	OPT Medication: Anti-infectives/Miscellaneous	0<br>1
opt_med_ANTIHISTAMINE AND DECONGESTANT COMBINATION	OPT Medication: Antihistamine and Decongestant Combination	0<br>1
opt_med_COLONY STIMULATING FACTORS	OPT Medication: Colony Stimulating Factors	0<br>1
opt_med_BLOOD	OPT Medication: Blood	0<br>1
opt_med_ANTI-OBESITY DRUGS	OPT Medication: Anti-Obesity Drugs	0<br>1
opt_med_ANALGESIC AND ANTIHISTAMINE COMBINATION	OPT Medication: Analgesic and Antihistamine Combination	0<br>1
opt_med_ANTIINFECTIVES	OPT Medication: Ant infectives	0<br>1
opt_med_ANTIINFLAM.TUMOR NECROSIS FACTOR INHIBITING AGENTS	OPT Medication: Anti-inflammatory & Tumor Necrosis Factor Inhibiting Agents	0<br>1
opt_med_ANTIALLERGY	OPT Medication: Antiallergy	0<br>1
opt_med_INVESTIGATIONAL	OPT Medication: Investigational	0<br>1
opt_med_BREAST MILK	OPT Medication: Breast Milk	0<br>1
opt_med_DIGITAL THERAPY	OPT Medication: Digital Therapy	0<br>1

eTable 3. Final List of Features Included in the Model and Associated Shapley Additive Explanations (SHAP) Values

Feature	Mean Absolute SHAP
Number of times restrained previously	0.782809
ESI Level 2	0.519132
ED Visits (Past Year)	0.442392
Presence of previous restraint	0.347534
Fall Risk Total Score	0.225217
ESI Level 4	0.221082
Chief Complaint: Psychiatric	0.218131
Chief Complaint: Substance Use Disorder	0.185569
Chief Complaint: Cardiovascular	0.14068
Chief Complaint: Musculoskeletal	0.125397
Chief Complaint: Respiratory	0.115008
Drug Type: Unknown	0.114077
Age	0.112957
Past Medical History: Symptoms of mental and substance use conditions	0.087289
Chief Complaint: Gastrointestinal	0.084557
Past Medical History: Psychotic conditions	0.078151
Current Smoking Status	0.077618
Respiratory Rate	0.076117
PULSE	0.076099
Chief Complaint: Miscellaneous	0.067757
Weight	0.067547
Chief Complaint: Procedural	0.06345
Chief Complaint: Neurological	0.058658
Chief Complaint: Infectious Diseases	0.04981
Chief Complaint: Agitation	0.044418
OPT Medication: Psychotherapeutic Drugs	0.044336
Past Medical History: Neurocognitive Disorders	0.041116
Systolic Blood Pressure	0.039744
Outpatient Medication: Antibiotics	0.033621
Past Medical History: Implant, Graft, or Device related encounter	0.033463
ESI Level 5	0.032015
Chief Complaint: Urological and Reproductive conditions	0.02625
Temperature	0.018101
Presence of Alcohol: Unknown	0.014869
SPO2	0.011025
Smoking Status: Unknown	0.009288
Presence of psychiatrist on treatment	0.004549
ESI Level 1	0.004537
Past Medical History: Other specified and unspecified disorders of stomach and duodenum	0.002691
Past Medical History: Socioeconomic/psychosocial factors	0.002397
Chief Complaint: Unknown (Unspecified)	0.002249
Past Medical History: Dysphagia	0.002109
Past Medical History: Refractive error	0.001445

© 2025 Wong AH et al. *JAMA Network Open*.

Past Medical History: Gastritis and duodenitis	0.000961
Past Medical History: Effect of foreign body entering opening, initial encounter	0.000833
Past Medical History: Erectile dysfunction	0.000787
Past Medical History: Sinusitis	0.000618
Past Medical History: Other specified female genital disorders	0.000422
Past Medical History: Outpatient Medication: Herbal	0.000376
Past Medical History: Diseases of mouth; excluding dental	0

© 2025 Wong AH et al. *JAMA Network Open*.

eTable 4. Model Evaluation Reports for Validation and External Datasets

Validation Dataset

Validation Accuracy: 0.9923548053398138

	Precision	Recall	F1 Score	Support
0	0.99	1.00	1.00	603414
1	0.86	0.20	0.33	5596
Accuracy			0.99	609010
Macro average	0.93	0.60	0.66	609010
Weighted average	0.99	0.99	0.99	609010

External Dataset

Validation Accuracy: 0.9923058179790385

	Precision	Recall	F1 Score	Support
0	0.99	1.00	1.00	605705
1	0.90	0.21	0.34	5796
Accuracy			0.99	611501
Macro average	0.94	0.61	0.67	611501
Weighted average	0.99	0.99	0.99	661501

© 2025 Wong AH et al. *JAMA Network Open*.

eTable 5. List of Sedative Medications and Percentages of Agitation Events That Included Administration of Each Medication

Sedative Medication Name	Percentage of Agitation Events with Sedative Administered
Haloperidol	35.5
Lorazepam	32.2
Diphenhydramine	13.3
Olanzapine	7.7
Droperidol	7.7
Midazolam	5.2
Ketamine	2.0
Aripiprazole	0.5
Hydroxyzine	0.3
Ziprasidone	0.2

© 2025 Wong AH et al. *JAMA Network Open*.

eTable 6. Fairness Assessment and Model Performance Across Age, Sex, and Race and Ethnicity Categories

	Training				Testing				External			
	AUROC (95% CI)	PR AUC (95% CI)	Calibration Intercept (95% CI)	Calibration Slope (95% CI)	AUROC (95% CI)	PR AUC (95% CI)	Calibration Intercept (95% CI)	Calibration Slope (95% CI)	AUROC (95% CI)	PR AUC (95% CI)	Calibration Intercept (95% CI)	Calibration Slope (95% CI)
**Age**												
18-45	0.968 (0.966 - 0.970)	0.510 (0.500 - 0.519)	-5.26 (-5.28- -5.23)	15.29 (14.99 - 15.61)	0.962 (0.958 - 0.965)	0.454 (0.437 - 0.469)	-5.11 (-5.15 - -5.07)	13.42 (12.95 - 13.93)	0.954 (0.950 - 0.957)	0.444 (0.425- 0.462)	-5.22 (-5.26 - -5.18)	14.06 (13.47 – 14.71)
46-65	0.959 (0.957 - 0.962)	0.416 (0.403 - 0.429)	-5.39 (-5.42 - -5.35)	17.88 (17.39 - 18.36)	0.948 (0.943 - 0.952)	0.352 (0.327 - 0.374)	-5.20 (-5.25 - -5.14)	14.66 (13.57 - 15.47)	0.929 (0.925 - 0.934)	0.351 (0.345- 0.351)	-5.16 (-5.22 - -5.11)	14.86 (13.97 – 15.79)
>65	0.946 (0.943 - 0.949)	0.405 (0.385 - 0.427)	-5.98 (-6.04 - -5.90)	17.83 (14.66 - 19.35)	0.923 (0.918 - 0.928)	0.347 (0.311 - 0.387)	-5.90 (-5.99 - -5.78)	11.03 (9.62 – 13.47)	0.917 (0.912 - 0.922)	0.415 (0.387- 0.442)	-5.08 (-5.14 - -5.01)	12.37 (11.24 - 14.12)
**Gender**												
Male	0.962 (0.959 - 0.964)	0.472 (0.462 - 0.481)	-5.21 (-5.23 - -5.19)	15.38 (15.05 - 15.69)	0.953 (0.949 - 0.956)	0.419 (0.403- 0.434)	-5.07 (-5.11 - -5.03)	13.26 (12.81 - 13.73)	0.936 (0.933 - 0.940)	0.414 (0.398- 0.429)	-4.95 (-4.99 - -4.91)	13.45 (12.74 – 14.20)
Female	0.965 (0.963 - 0.967)	0.467 (0.455 - 0.478)	-5.68 (-5.71 - -5.65)	17.59 (17.14 - 18.06)	0.955 (0.951 - 0.958)	0.399 (0.381- 0.419)	-5.56 (-5.62 - -5.51)	15.44 (14.51 - 16.32)	0.940 (0.936 - 0.943)	0.395 (0.376- 0.413)	-5.41 (-5.46 - -5.37)	15.49 (14.65 – 16.36)
**Race**												
White	0.963 (0.962 - 0.965)	0.472 (0.463 - 0.481)	-5.52 (-5.58 - -5.49)	17.17 (16.50 – 18.13)	0.953 (0.950 - 0.956)	0.413 (0.396 - 0.429)	-5.39 (-5.43 - -5.34)	14.97 (14.31 - 15.56)	0.931 (0.927 - 0.934)	0.410 (0.391- 0.429)	-5.05 (-5.10 - -5.00)	13.99 (13.03 – 14.84)
Black	0.960 (0.957 - 0.963)	0.431 (0.417 - 0.444)	-5.19 (-5.22 - -5.15)	15.76 (15.32 – 16.21)	0.951 (0.946 - 0.957)	0.365 (0.342- 0.389)	-5.05 (-5.11 - -4.99)	13.55 (12.70 - 14.32)	0.943 (0.938 - 0.947)	0.397 (0.374- 0.416)	-5.06 (-5.12 - -5.01)	13.82 (13.00 – 14.73)
Asian	0.989 (0.977 – 1.000)	0.635 (0.559 - 0.711)	-5.81 (-5.99 - -5.66)	10.67 (9.33 – 12.35)	0.970 (0.950 – 0.990)	0.594 (0.446- 0.739)	-5.68 (-6.00 - -5.40)	7.00 (6.06 - 7.77)	0.916 (0.889 - 0.943)	0.516 (0.313- 0.712)	-5.51 (-5.91 - -5.18)	5.49 (4.16 - 6.34)
American Indian/Native American	0.953 (0.931 - 0.974)	0.393 (0.284 - 0.503)	-4.98 (-5.22 - -4.75)	6.79 (6.02 - 7.58)	0.965 (0.928 - 1.003)	0.475 (0.275- 0.654)	-4.86 (-5.31 - -4.48)	3.85 (1.97 - 5.26)	0.899 (0.846 - 0.951)	0.425 (0.161- 0.661)	-4.59 (-5.18 - -4.16)	3.45 (1.43 - 4.67)
Native Hawaiian/ Pacific Islander	0.959 (0.935 - 0.983)	0.337 (0.209 - 0.473)	-4.99 (-5.27 - -4.75)	6.27 (4.98 - 7.66)	0.979 (0.937 - 1.021)	0.491 (0.204 - 0.732)	-5.12 (-5.75 - -4.70)	3.84 (1.43 - 5.22)	0.986 (0.938 - 1.034)	0.392 (0.090 - 0.698)	-5.21 (-6.05 - -4.66)	2.28 (0.47 - 4.07)
Other	0.969 (0.964 - 0.973)	0.466 (0.442 - 0.490)	-5.50 (-5.56 - -5.44)	15.39 (14.55 – 16.18)	0.961 (0.953 – 0.968)	0.401 (0.362 – 0.439)	-5.28 (-5.37 - -5.19)	12.12 (11.08 – 13.37)	0.944 (0.940 – 0.949)	0.378 (0.351 – 0.404)	-5.38 (-5.45 - -5.32)	14.15 (13.12 – 15.23)
Unknown	0.982 (0.974 - 0.990)	0.686 (0.651 - 0.723)	-5.50 (-5.61 - -5.39)	13.00 (11.69 – 14.45)	0.982 (0.968 – 0.996)	0.653 (0.585 – 0.714)	-5.30 (-5.48 - -5.13)	8.98 (8.39 – 9.67)	0.971 (0.959 – 0.984)	0.612 (0.537 – 0.692)	-5.75 (-5.97 - -5.55)	8.14 (7.83 – 8.57)
**Ethnicity**												
Non-Hispanics	0.963 (0.961 - 0.964)	0.463 (0.455 - 0.471)	-5.43 (-5.45 - -5.40)	16.73 (16.43 – 17.10)	0.954 (0.951 - 0.956)	0.406 (0.392- 0.421)	-5.32 (-5.36 - -5.28)	14.90 (14.33 - 15.48)	0.936 (0.933 - 0.939)	0.403 (0.389- 0.417)	-5.06 (-5.09 - -5.03)	14.34 (13.69 – 14.91)
Hispanics	0.970 (0.966 - 0.973)	0.472 (0.453 - 0.490)	-5.53 (-5.58 - -5.48)	15.82 (15.13 - 16.55)	0.962 (0.956 - 0.968)	0.402 (0.371- 0.432)	-5.31 (-5.38 - -5.23)	13.14 (12.20 – 14.25)	0.945 (0.941 - 0.949)	0.410 (0.383- 0.436)	-5.45 (-5.51 - -5.39)	14.64 (13.19 – 15.67)
Unknown	0.991 (0.975 – 1.007)	0.839 (0.787 – 0.886)	-5.58 (-5.85 - -5.37)	8.86 (8.55 – 9.29)	0.971 (0.944 – 0.999)	0.763 (0.672 – 0.851)	-5.09 (-5.40 - -4.79)	7.09 (6.67 – 7.56)	0.978 (0.943 – 1.014)	0.735 (0.557 – 0.913)	-5.48 (-5.87 - -5.09)	5.81 (4.78 – 6.49)

© 2025 Wong AH et al. *JAMA Network Open*.

eFigure. Model Performance During Cross-Validation. This figure demonstrates area under the receiver operating characteristic curve (AUROC curve), area under Precision-Recall Curves (PR-AUC curve), and the confusion matrix for the prediction model during cross-validation.

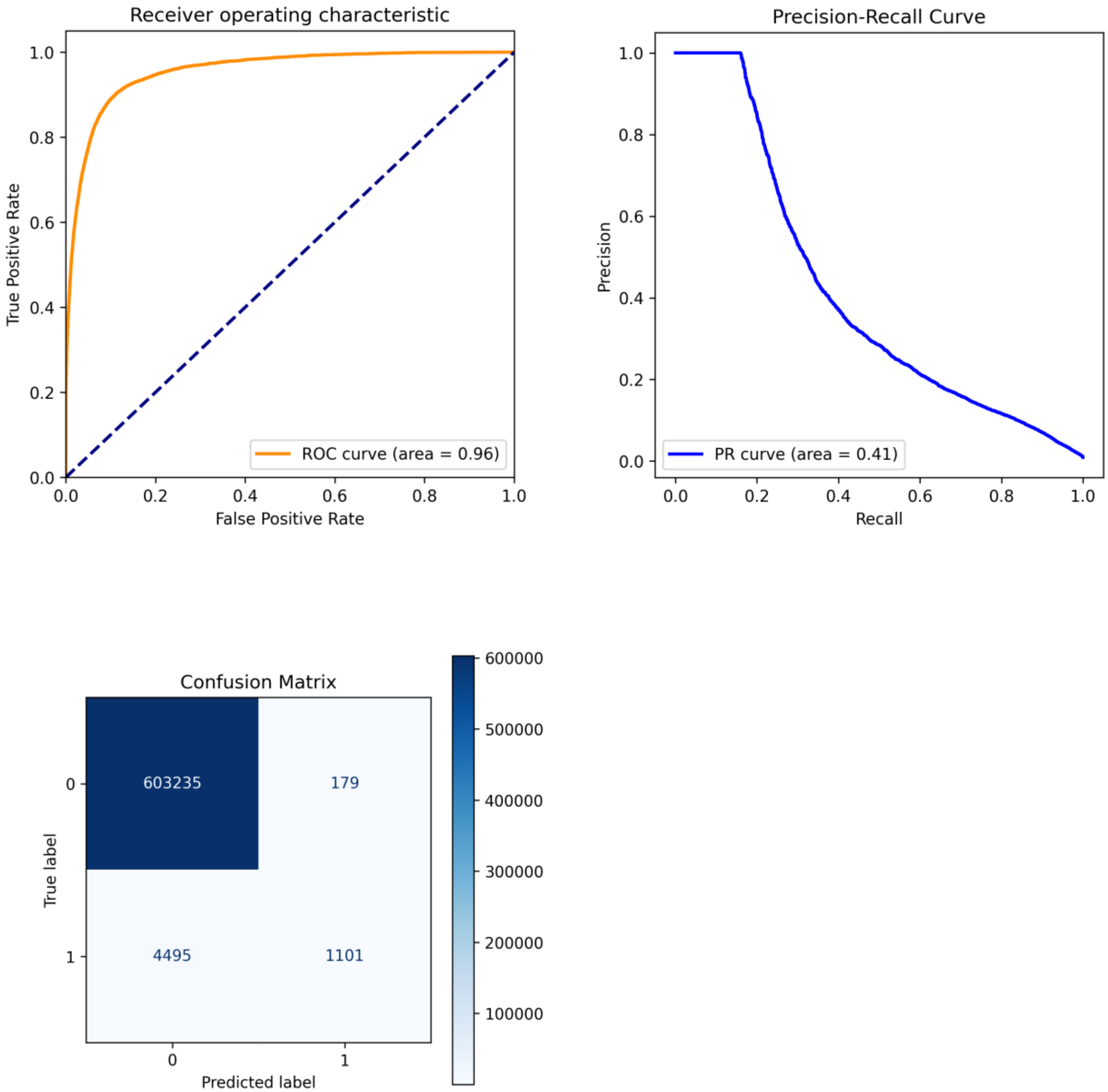

© 2025 Wong AH et al. *JAMA Network Open*.
