# Supplementary material for: Predicting Agitation Events in the Emergency Department Through Artificial Intelligence
Source: JAMA Netw Open. 2025 May 7;8(5):e258927. doi: 10.1001/jamanetworkopen.2025.8927 (PMC12059975; doi:10.1001/jamanetworkopen.2025.8927)
Supplement: Supplement 2. — Data Sharing Statement [file jamanetwopen-e258927-s002.pdf]

Data Sharing Statement

Wong. Predicting Agitation Events in the Emergency Department Through Artificial Intelligence. *JAMA Netw Open*. Published May 07, 2025. doi:10.1001/jamanetworkopen.2025.8927

Data

Data available: Deidentified formats of the data are available for request via Yale University School of Medicine Institutional Data Access and Ethics Committee for academic researchers who meet the committee's criteria for access to the institution's confidential EHR data through the creation of appropriate data use agreements (https://privacy.yale.edu/resources/sharing-data).
